# Supplementary material for: Machine learning-based urban noise appropriateness evaluation method and driving factor analysis
Source: PLoS One. 2024 Dec 23;19(12):e0311571. doi: 10.1371/journal.pone.0311571 (PMC11666067; doi:10.1371/journal.pone.0311571)
Supplement: S1 Data — (ZIP) [file pone.0311571.s001.zip › supporting data/2023 Monitoring Report of Jiangxi Ecological Environment Monitoring Center.pdf]

江 西 省 生 态 环 境 监 测 中 心

监 测 报 告

赣环监字（2023）第 NC-LX088 号

项目名称： 2023 年南昌市道路交通噪声例行监测

委托单位： 江西省生态环境监测中心

监测类别： 例　行

报告日期： 2023 年 10 月 25 日

（加盖检验检测专用章）

江 西 省 生 态 环 境 监 测 中 心

检测场所地址：江西省南昌市红谷滩区怡园路 1166 号

监测报告说明

1、本报告无CMA专用章、本中心检验检测专用章和骑缝章无效。

2、报告内容需填写齐全，无审核、签发者签字无效。

3、报告需填写清楚，涂改无效。

4、监测委托方如对监测报告有异议，须于收到本监测报告之日起十日内向我中心提出，逾期不予受理。无法保存、复现的样品不受理申诉。

5、由委托单位自行采集的样品，仅对送检样品检测数据负责，不对样品来源负责。

6、本报告未经同意不得用于广告宣传。

7、复制本报告中的部分内容无效。

单位地址：江西省南昌市红谷滩区怡园路1166号

邮政编码：330038

电　　话：0791-86356075

传　　真：0791-86356077

电子邮件：jcz@lsnc.cn

联 系 人：王瑶瑶

1、监测项目概况

1.1 项目名称：2023 年南昌市道路交通噪声例行监测。

1.2 监测内容：根据 2023 年江西省生态环境监测方案，江西省南昌生态环境监测中心开展了 2023 年南昌市道路交通噪声（昼间、夜间）例行监测工作。

1.3 监测人员：徐义邦、万磊、张郸、邓娟华、宋涓、熊小宝、肖军、盛寋、罗松、胡晓珍、章健、章美文、熊俊龙、帅俊松、张瞳、万鹏、魏信逵、彭存荣、于鹏宏、罗政博、高建亮、徐义邦、王一迪、杨哲欣、曾湘静。

1.4 采样时间：2023 年 2 月 14 日-9 月 27 日。

2、监测项目及安排

表 1 噪声检测方法及监测仪器一览表

项目名称	检测方法	仪器名称、型号	最低检出限	仪器编号	监测人员
交通噪声	（1）《环境噪声监测技术规范 城市声环境常规监测》（HJ 640-2012）<br>（2）《声环境质量标准》（GB3096-2008）	1、多功能声级计（I）AWA6228+<br>2、多功能声级计（I）AWA6228	/	JC-1076<br>JC-1077<br>JC-1264<br>JC-1265<br>JC-1315<br>JC-1316<br>JC-1317<br>JC-1318<br>JC-1319<br>JC-JS030<br>JC-JS048-7	万磊、张郸、邓娟华、宋涓、熊小宝、肖军、盛寋、罗松、胡晓珍、章健、章美文、熊俊龙、帅俊松、张瞳、万鹏、魏信逵、彭存荣、于鹏宏、罗政博、高建亮、徐义邦、王一迪、杨哲欣、曾湘静

表 2 噪声监测点位、频次一览表

监测单位	监测点位	GPS 信息	监测项目	监测频次	采样人员
江西省南昌生态环境监测中心	见表 3、表 4 各监测点位	见表 3、表 4 各监测点位	交通噪声	20 分钟 1 点/1 次	万磊、张郸、邓娟华、宋涓、熊小宝、肖军、盛寋、罗松、胡晓珍、章健、章美文、熊俊龙、帅俊松、张瞳、万鹏、魏信逵、彭存荣、于鹏宏、罗政博、高建亮、徐义邦、王一迪、杨哲欣、曾湘静

3、监测结果

昼间监测结果见表 3，夜间监测结果见表 4。

表3　2023年南昌市道路交通噪声昼间例行监测

行政区划代码	监测年度	点位编码	测点名称	测点经度	测点纬度	路段名称	路段长度（m）	道路总宽度（m）	机动车车道数	车道类别	道路等级	测点参照物	路段覆盖人口（万人）	月	日	时	分	大型车车流量	中小型车车流量	Leq	L10	L50	L90	最大值	最小值	标准差(SD)	监测站名	监测仪器型号	监测仪器编号	监测前校准值	监测后校准值	声校准器测量声压值	声校准仪器型号	声校准仪器编号
360100	2023	360111200118	艾溪湖北路 I	115.9950	28.7130	艾溪湖北路	6965	30	4	2	2	南昌市高新区中兴和园	0.3572	8	21	11	13	19	148	66.7	69.4	63.4	54.8	95.1	43.3	6.0	江西省南昌生态环境监测中心	AWA6228+	JC-1076	93.8	93.5	94.0	AWA6223-F	FZ-0735
360100	2023	360103200062	八一大道 II 号	115.9005	28.6699	八一大道	3031	50	10	2	2	南昌市西湖区赣江宾馆	0.2425	8	23	17	1	64	1247	67.5	69.4	67.2	63.8	80.0	59.6	2.2	江西省南昌生态环境监测中心	AWA6228+	JC-1317	93.8	93.9	94.0	AWA6223-F	FZ-0737
360100	2023	360103200096	北京路 I 号	115.9096	28.6771	北京西路	2171	41	6	2	2	南昌市西湖区工商银行北京西路支行	0.1737	8	22	11	23	21	801	69.4	71.2	67.6	62.4	93.0	55.2	3.7	江西省南昌生态环境监测中心	AWA6228+	JC-1265	93.8	93.6	94.0	AWA6223-F	FZ-0737
360100	2023	360111200098	北京路III号	115.9497	28.6779	北京东路	4821	48	6	2	2	南昌市青山湖区江西省肿瘤医院	0.3857	8	22	9	58	12	1280	69.1	69.4	64.6	62.0	92.7	57.6	3.7	江西省南昌生态环境监测中心	AWA6228+	JC-1315	93.8	93.5	94.0	AWA6223-F	FZ-0735
360100	2023	360111200112	昌东大道 I 号	115.9939	28.7005	昌东大道	5631	77	6	2	2	南昌市高新区武警水电二路总队医院	0.2779	8	21	10	45	34	489	62.9	64.2	59.8	56.6	87.5	53.1	4.0	江西省南昌生态环境监测中心	AWA6228	JC-JS030	93.8	94.1	94.0	AWA6223-F	FZ-0735
360100	2023	360111200114	昌东大道III号	115.9755	28.6484	昌东大道	4087	36	4	2	2	南昌市青山湖区城东花园	0.223	8	21	15	30	173	928	69.4	72.0	67.8	60.2	88.7	54.4	4.7	江西省南昌生态环境监测中心	AWA6228+	JC-1076	93.8	93.5	94.0	AWA6223-F	FZ-0735
360100	2023	360111200115	昌东大道IV号	115.9746	28.6185	昌东大道	5264	36	6	2	2	南昌市青山湖区雪津南昌啤酒公司	0.4211	8	21	16	24	60	1800	70.9	71.8	66.0	57.2	108.8	31.0	5.7	江西省南昌生态环境监测中心	AWA6228+	JC-1315	93.8	93.9	94.0	AWA6223-F	FZ-0735
360100	2023	360103200109	昌南大道 I 号	115.8651	28.6158	昌南大道	6465	40	6	2	2	南昌市西湖区象湖水产场	0.117	8	23	14	2	77	1578	73.1	75.4	72.0	68.8	84.5	63.5	2.7	江西省南昌生态环境监测中心	AWA6228+	JC-1318	93.8	93.9	94.0	AWA6223-F	FZ-0737
360100	2023	360111200111	昌南大道III号	115.9423	28.6007	昌南大道	6569	52	6	2	2	南昌市青山湖区京川村	0.2922	8	23	11	16	28	752	68.0	70.2	65.7	60.8	87.9	57.1	4.6	江西省南昌生态环境监测中心	AWA6228	JC-JS030	93.8	93.6	94.0	AWA6223-F	FZ-0735
360100	2023	360111200015	丰和大道 II 号	115.8697	28.7086	丰和北大道	3844	45	6	2	2	南昌市红谷滩区南昌日报社	0.2334	8	22	16	3	18	410	53.2	56.2	49.8	41.2	71.2	36.8	5.7	江西省南昌生态环境监测中心	AWA6228+	JC-1077	93.8	93.7	94.0	AWA6223-F	FZ-0736
360100	2023	360111200016	丰和大道III号	115.8533	28.6964	丰和中大道	4217	56	6	2	2	南昌市红谷滩区丰和小区	0.3374	8	22	15	30	35	338	57.1	58.6	53.8	47.8	84.3	40.7	4.4	江西省南昌生态环境监测中心	AWA6228+	JC-1077	93.8	93.7	94.0	AWA6223-F	FZ-0736
360100	2023	360112200017	丰和大道IV号	115.8261	28.6461	丰和南大道	5327	56	6	2	2	南昌市红谷滩区中环医院	0.2841	8	23	9	50	18	480	56.7	60.6	50.4	41.2	77.3	32.0	7.8	江西省南昌生态环境监测中心	AWA6228+	JC-1077	93.8	93.6	94.0	AWA6223-F	FZ-0736
360100	2023	360103200052	抚河路 I 号	115.8794	28.6760	抚河北路	1536	24	6	2	3	南昌市西湖区独一处粥城	0.1229	8	23	10	27	22	398	70.4	73.6	68.6	56.8	89.6	45.8	6.4	江西省南昌生态环境监测中心	AWA6228+	JC-1265	93.8	93.7	94.0	AWA6223-F	FZ-0737
360100	2023	360103200053	抚河路 II 号	115.8879	28.6674	抚河中路	1707	24	6	2	3	南昌市西湖区永新汽车装璜维修中心	0.1366	8	23	10	26	23	461	69.4	72.8	68.0	60.4	83.8	52.9	4.6	江西省南昌生态环境监测中心	AWA6228+	JC-1318	93.8	93.9	94.0	AWA6223-F	FZ-0737
360100	2023	360104200054	抚河路III号	115.8961	28.6523	抚河南路	2369	30	6	2	2	南昌市青云谱区省建材大市场	0.1895	8	23	16	25	17	503	70.4	73.2	69.6	64.6	87.2	55.9	3.4	江西省南昌生态环境监测中心	AWA6228+	JC-1265	93.8	93.7	94.0	AWA6223-F	FZ-0737
360100	2023	360103200041	抚生路 I 号	115.8742	28.6627	抚生路	1028	31	4	2	3	南昌市西湖区卓品骄阳住宅小区	0.0822	8	23	9	10	2	258	64.7	66.6	61.8	56.8	88.7	52.0	3.9	江西省南昌生态环境监测中心	AWA6228+	JC-1318	93.8	93.8	94.0	AWA6223-F	FZ-0737
360100	2023	360103200042	抚生路 II 号	115.8664	28.6491	抚生南路	2508	31	4	2	3	南昌市西湖区大塘村农民公寓	0.2006	8	23	16	4	26	385	67.4	69.8	64.8	60.2	86.6	53.2	4.0	江西省南昌生态环境监测中心	AWA6228+	JC-1318	93.8	93.9	94.0	AWA6223-F	FZ-0737
360100	2023	360103200043	抚生路III号	115.8534	28.6291	抚生南路	2710	31	4	2	3	南昌市西湖区抚生路小学	0.1084	8	23	14	21	8	120	63.9	68.0	60.8	50.4	80.4	44.4	6.5	江西省南昌生态环境监测中心	AWA6228+	JC-1265	93.8	93.9	94.0	AWA6223-F	FZ-0737
360100	2023	360111200020	赣江大道 I 号	115.8801	28.7119	赣江北大道	3409	18	4	2	2	南昌市红谷滩区绿宝竹木业公司	0.0884	8	22	16	11	3	81	64.2	64.6	58.2	50.6	92.5	45.5	5.3	江西省南昌生态环境监测中心	AWA6228+	JC-1319	93.8	93.7	94.0	AWA6223-F	FZ-0736
360100	2023	360102200021	赣江大道 II 号	115.8625	28.6915	赣江中大道	4007	36	6	2	2	南昌市红谷滩区南昌广播电视中心	0.1603	8	22	15	1	5	99	63.1	65.8	62.4	56.4	78.8	49.6	3.7	江西省南昌生态环境监测中心	AWA6228+	JC-1319	93.8	93.9	94.0	AWA6223-F	FZ-0736
360100	2023	360112200023	赣江大道IV号	115.8473	28.6618	赣江南大道	5379	40	4	2	2	南昌市红谷滩区赣江新天地网球场	0.161	8	22	14	9	10	275	56.9	61.0	53.2	44.6	75.0	38.7	6.1	江西省南昌生态环境监测中心	AWA6228+	JC-1077	93.8	93.7	94.0	AWA6223-F	FZ-0736
360100	2023	360111200082	高新大道 II 号	115.9561	28.7025	高新大道	6104	50	6	2	2	南昌市青山湖区江西天一药业公司	0.3454	8	22	11	7	24	536	61.9	64.5	60.1	54.8	80.2	48.3	4.1	江西省南昌生态环境监测中心	AWA6228	JC-JS030	93.8	94.0	94.0	AWA6223-F	FZ-0735
360100	2023	360111200086	高新大道VI号	115.9518	28.6353	高新南大道	8837	50	6	2	2	南昌市青山湖区石桥村	0.4579	8	21	16	35	21	475	61.2	63.8	60.0	56.4	76.9	51.9	3.0	江西省南昌生态环境监测中心	AWA6228	JC-JS030	93.8	94.2	94.0	AWA6223-F	FZ-0735
360100	2023	360104200108	广州路 II 号	115.9435	28.6233	广州路	5946	39	4	2	2	南昌市青山湖区万溪村	0.3363	8	23	14	8	31	688	64.3	66.8	62.6	59.0	82.5	55.1	3.8	江西省南昌生态环境监测中心	AWA6228	JC-JS030	93.8	93.6	94.0	AWA6223-F	FZ-0735
360100	2023	360103200048	洪城路 II 号	115.8871	28.6586	洪城路	3909	34	8	2	2	南昌市西湖区东方巴黎大厦	0.3127	8	23	16	56	77	1144	70.2	72.6	69.2	65.6	87.6	62.8	2.7	江西省南昌生态环境监测中心	AWA6228+	JC-1318	93.8	94.1	94.0	AWA6223-F	FZ-0737
360100	2023	360102200067	洪都大道 I 号	115.9177	28.6987	洪都北大道	4463	44	6	2	2	南昌市东湖区江西省地震局	0.357	8	22	9	22	16	361	67.7	70.4	66.2	62.8	82.2	58.8	3.0	江西省南昌生态环境监测中心	AWA6228+	JC-1265	93.8	93.9	94.0	AWA6223-F	FZ-0737
360100	2023	360103200070	洪都大道IV号	115.9225	28.6632	洪都中大道	2245	44	6	2	2	南昌市青山湖区隆鑫广场小区	0.1796	8	22	15	56	22	326	66.4	69.0	65.1	62.0	80.1	59.0	3.0	江西省南昌生态环境监测中心	AWA6228	JC-JS030	93.8	94.1	94.0	AWA6223-F	FZ-0735
360100	2023	360111200095	火炬大街 I 号	115.9601	28.6942	火炬大街	2815	35	4	2	2	南昌市青山湖区百大集团物流中心	0.2252	8	22	10	57	43	526	66.3	68.6	65.0	59.8	84.2	52.8	3.5	江西省南昌生态环境监测中心	AWA6228+	JC-1076	93.8	93.7	94.0	AWA6223-F	FZ-0735
360100	2023	360111200104	解放路 II 号	115.9357	28.6554	解放西路	3993	35	6	2	2	南昌市青山湖区南昌公交修理二厂	0.3194	8	22	17	0	43	1483	62.4	57.0	50.8	48.8	89.3	46.6	5.1	江西省南昌生态环境监测中心	AWA6228+	JC-1076	93.8	93.6	94.0	AWA6223-F	FZ-0735
360100	2023	360111200106	解放路IV号	115.9638	28.6449	解放东路	4976	42	4	2	2	南昌市青山湖区沈桥村	0.3981	8	24	10	39	15	265	64.4	66.6	61.9	57.0	87.1	52.9	4.8	江西省南昌生态环境监测中心	AWA6228	JC-JS030	93.8	93.4	94.0	AWA6223-F	FZ-0735
360100	2023	360111200025	锦江路 I 号	115.8724	28.7179	锦江路	1432	16	4	2	3	南昌市红谷滩区凤凰花园	0.1146	8	22	16	30	0	50	45.8	48.8	39.0	32.2	73.2	26.1	6.3	江西省南昌生态环境监测中心	AWA6228+	JC-1077	93.8	93.7	94.0	AWA6223-F	FZ-0736
360100	2023	360111200089	京东大道 II 号	115.9683	28.7069	京东大道	6442	40	4	2	2	南昌市青山湖区江西丰采丽印刷包装公司	0.5154	8	22	14	49	12	180	61.2	64.1	59.7	53.0	85.6	48.9	4.7	江西省南昌生态环境监测中心	AWA6228	JC-JS030	93.8	94.1	94.0	AWA6223-F	FZ-0735
360100	2023	360111200091	京东大道IV号	115.9627	28.6685	京东南大道	3559	40	4	2	2	南昌市青山湖区世纪风情住宅小区	0.1924	8	21	14	40	9	390	63.0	65.7	62.1	56.4	79.4	49.7	4.4	江西省南昌生态环境监测中心	AWA6228	JC-JS030	93.8	93.4	94.0	AWA6223-F	FZ-0735
360100	2023	360104200064	井冈山大道 II	115.9069	28.6489	井冈山大道	4692	50	8	2	2	南昌市青云谱区深圳农产品批发市场	0.3754	8	23	9	45	72	847	66.2	68.2	63.6	58.0	90.7	50.4	4.2	江西省南昌生态环境监测中心	AWA6228+	JC-1076	93.8	93.6	94.0	AWA6223-F	FZ-0735
360100	2023	360103200050	九洲大街 I 号	115.8580	28.6415	九洲大街	1366	44	6	2	3	南昌市西湖区观洲村	0.1093	8	23	15	41	6	765	68.2	71.4	66.8	63.2	80.8	59.4	3.0	江西省南昌生态环境监测中心	AWA6228+	JC-1317	93.8	93.9	94.0	AWA6223-F	FZ-0737
360100	2023	360103200051	九洲大街 II 号	115.8706	28.6319	九洲大街	2077	44	6	2	3	南昌市西湖区南昌动物园	0.1662	8	23	14	48	13	522	65.9	68.8	63.8	56.6	86.7	49.4	4.8	江西省南昌生态环境监测中心	AWA6228+	JC-1318	93.8	93.8	94.0	AWA6223-F	FZ-0737
360100	2023	360111200010	庐山南大道 I	115.8461	28.7366	庐山南大道	1964	46	4	2	2	南昌市青山湖区昌北电信分公司	0.1571	8	21	14	31	72	487	52.9	55.6	51.2	46.4	74.6	38.8	3.7	江西省南昌生态环境监测中心	AWA6228+	JC-1077	93.8	94.0	94.0	AWA6223-F	FZ-0736
360100	2023	360111200012	庐山南大道III	115.8594	28.7063	庐山南大道	3865	35	4	2	2	南昌市红谷滩区工商银行昌北支行	0.3092	8	22	15	39	56	148	66.9	68.6	64.8	59.4	88.4	53.1	3.7	江西省南昌生态环境监测中心	AWA6228+	JC-1319	93.8	93.7	94.0	AWA6223-F	FZ-0736

表3　2023年南昌市道路交通噪声昼间例行监测

行政区划代码	监测年度	点位编码	测点名称	测点经度	测点纬度	路段名称	路段长度（m）	道路总宽度（m）	机动车车道数	车道类别	道路等级	测点参照物	路段覆盖人口（万人）	月	日	时	分	大型车车流量	中小型车车流量	Leq	L10	L50	L90	最大值	最小值	标准差(SD)	监测站名	监测仪器型号	监测仪器编号	监测前校准值	监测后校准值	声校准器测量声压值	声校准仪器型号	声校准仪器编号
360100	2023	360104200066	南莲路Ⅰ号	115.9183	28.6140	南莲路	2439	32	4	2	2	南昌市青云谱区熊坊小学	0.1951	8	23	10	56	27	387	69.8	72.8	68.6	62.2	91.9	54.0	4.0	江西省南昌生态环境监测中心	AWA6228^{+}	JC-1076	93.8	93.6	94.0	AWA6223-F	FZ-0735
360100	2023	360112200029	南斯友好路Ⅰ	115.8348	28.6749	南斯友好路	3132	24	4	2	2	南昌市红谷滩区省二手车市场	0.2506	8	22	14	23	58	241	75.3	78.4	74.2	68.0	93.5	61.1	3.9	江西省南昌生态环境监测中心	AWA6228^{+}	JC-1319	93.8	93.7	94.0	AWA6223-F	FZ-0736
360100	2023	360112200034	前湖大道Ⅰ号	115.8214	28.6612	前湖大道	4034	82	6	2	2	南昌市红谷滩区南昌航空大学红角洲校区	0.2656	8	23	10	1	8	146	72.2	74.8	66.4	60.2	90.8	57.7	5.8	江西省南昌生态环境监测中心	AWA6228^{+}	JC-1319	93.8	93.9	94.0	AWA6223-F	FZ-0736
360100	2023	360111200007	青岚大道Ⅰ号	115.8199	28.7505	青岚大道	1651	16	4	2	3	南昌市青山湖区昌北经济适用房	0.066	8	21	11	31	15	53	60.8	63.4	56.8	51.2	84.3	47.7	4.9	江西省南昌生态环境监测中心	AWA6228^{+}	JC-1319	93.8	93.9	94.0	AWA6223-F	FZ-0736
360100	2023	360111200008	青岚大道Ⅱ号	115.8198	28.7351	青岚大道	3466	16	4	2	3	南昌市青山湖区江西三星气龙公司	0.2053	8	21	14	28	22	103	68.8	68.0	56.8	49.0	99.4	44.0	7.3	江西省南昌生态环境监测中心	AWA6228^{+}	JC-1316	93.8	93.5	94.0	AWA6223-F	FZ-0736
360100	2023	360102200059	青山路Ⅰ号	115.9243	28.7138	青山北路	2288	22	4	2	3	南昌市东湖区江西强力工量刃具公司	0.183	8	22	8	54	16	317	65.9	69.6	63.6	56.8	82.0	51.2	4.7	江西省南昌生态环境监测中心	AWA6228^{+}	JC-1318	93.8	93.6	94.0	AWA6223-F	FZ-0737
360100	2023	360102200060	青山路Ⅱ号	115.9086	28.7028	青山南路	2632	35	6	2	2	南昌市东湖区百货大楼青山路店	0.2106	8	22	9	7	43	378	69.2	71.4	68.0	64.0	87.8	57.0	3.1	江西省南昌生态环境监测中心	AWA6228^{+}	JC-1317	93.8	93.9	94.0	AWA6223-F	FZ-0737
360100	2023	360103200074	孺子路Ⅰ号	115.8849	28.6748	孺子路	776	23	4	2	3	南昌市西湖区南昌华云中医院	0 0621	8	22	15	21	8	347	68.8	71.4	67.2	60.6	90.4	52.8	4.3	江西省南昌生态环境监测中心	AWA6228^{+}	JC-1317	93.8	93.8	94.0	AWA6223-F	FZ-0737
360100	2023	360103200075	孺子路Ⅱ号	115.8932	28.6751	孺子路	1020	25	4	2	3	南昌市西湖区金世家橱柜	0.0816	8	22	14	36	7	321	68.8	71.8	67.2	60.8	87.9	53.4	4.3	江西省南昌生态环境监测中心	AWA6228^{+}	JC-1317	93.8	94.0	94.0	AWA6223-F	FZ-0737
360100	2023	360104200077	三店西路Ⅱ号	115.9061	28.6338	三店西路	1503	20	4	2	3	南昌市青云谱区青云明珠小区	0.1202	8	24	10	14	6	186	65.5	67.6	64.0	60.2	85.1	55.0	3.1	江西省南昌生态环境监测中心	AWA6228^{+}	JC-1315	93.8	93.9	94.0	AWA6223-F	FZ-0735
360100	2023	360111200078	上海路Ⅰ号	115.9356	28.6880	上海北路	2771	20	4	2	3	南昌市青山湖区南昌大学附属中学	0.2217	8	22	9	44	23	252	61.8	64.6	59.6	53.2	81.0	45.5	4.5	江西省南昌生态环境监测中心	AWA6228^{+}	JC-1076	93.8	93.6	94.0	AWA6223-F	FZ-0735
360100	2023	360111200079	上海路Ⅱ号	115.9361	28.6705	上海路	2438	29	4	2	3	南昌市青山湖区南昌新时代妇科医院	0.195	8	22	10	11	10	175	64.9	66.9	63.2	57.8	92.9	52.3	4.1	江西省南昌生态环境监测中心	AWA6228	JC-JS030	93.8	94.0	94.0	AWA6223-F	FZ-0735
360100	2023	360102200028	世贸路Ⅱ号	115.8525	28.6853	世贸路	2036	18	4	2	3	南昌市红谷滩区南昌市人民政府	0.1629	8	22	14	54	10	168	58.3	60.4	53.4	47.2	91.6	40.8	5.0	江西省南昌生态环境监测中心	AWA6228^{+}	JC-1077	93.8	93.7	94.0	AWA6223-F	FZ-0736
360100	2023	360111200002	双港大街Ⅱ号	115.8312	28.7456	双港西大街	3884	32	6	2	2	南昌市青山湖区神基科技南昌公司	0.2227	8	21	11	25	12	138	62.8	66.0	60.6	56.2	80.7	50.9	3.7	江西省南昌生态环境监测中心	AWA6228^{+}	JC-1316	93.8	93.5	94.0	AWA6223-F	FZ-0736
360100	2023	360111200003	双港大街Ⅲ号	115.8647	28.7464	双港东大街	4175	50	6	2	2	南昌市青山湖区华东交大南区专家楼	0.1918	8	21	11	10	21	138	61.0	65.4	55.0	47.4	81.3	42.3	6.6	江西省南昌生态环境监测中心	AWA6228^{+}	JC-1077	93.8	93.9	94.0	AWA6223-F	FZ-0736
360100	2023	360111200101	顺外路Ⅱ号	115.9481	28.6649	顺外路	5342	17	4	2	2	南昌市青山湖区肖坊村民委员会	0.4274	8	22	16	10	12	370	64.2	64.2	60.0	55.4	95.6	50.0	3.4	江西省南昌生态环境监测中心	AWA6228^{+}	JC-1076	93.8	93.6	94.0	AWA6223-F	FZ-0735
360100	2023	360103200044	桃花路Ⅰ号	115.8790	28.6622	桃花路	1138	19	4	2	3	南昌市西湖区远东国际花园	0.091	8	23	9	48	22	387	68.8	72.0	67.4	59.0	86.1	52.8	4.8	江西省南昌生态环境监测中心	AWA6228^{+}	JC-1317	93.8	93.9	94.0	AWA6223-F	FZ-0737
360100	2023	360103200045	桃花路Ⅱ号	115.8764	28.6422	桃花南路	3209	38	6	2	3	南昌市西湖区坝上新村	0.1938	8	23	9	34	11	287	71.2	71.4	66.4	60.0	92.9	52.9	5.0	江西省南昌生态环境监测中心	AWA6228^{+}	JC-1265	93.8	93.9	94.0	AWA6223-F	FZ-0737
360100	2023	360103200046	桃花路Ⅲ号	115.8667	28.6233	桃花南路	1940	38	6	2	3	南昌市西湖区十里庙村	0.1552	8	23	14	26	4	1126	67.5	69.6	67.2	63.6	78.7	58.0	2.3	江西省南昌生态环境监测中心	AWA6228^{+}	JC-1317	93.8	93.9	94.0	AWA6223-F	FZ-0737
360100	2023	360121200116	天祥大道Ⅰ号	116.0219	28.6982	天祥大道	5830	21	4	2	2	南昌市高新区南昌工业职业技术学院	0.3413	8	21	10	9	27	281	67.2	71.0	62.4	55.6	82.7	49.5	5.7	江西省南昌生态环境监测中心	AWA6228^{+}	JC-1317	93.8	94.0	94.0	AWA6223-F	FZ-0737
360100	2023	360112200036	祥云大道Ⅰ号	115.8030	28.6356	祥云大道	8314	48	6	2	2	南昌市红谷滩区中铁16局4公司南昌项目部	0.4047	8	23	9	10	808	2981	52.6	54.6	50.8	47.4	77.0	42.2	3.0	江西省南昌生态环境监测中心	AWA6228^{+}	JC-1077	93.8	93.6	94.0	AWA6223-F	FZ-0736
360100	2023	360102200057	象山路Ⅰ号	115.8861	28.6825	象山北路	1367	18	4	2	3	南昌市东湖区南昌市第一医院	0.1094	8	22	14	54	16	319	65.0	67.0	62.8	58.6	87.2	53.0	3.6	江西省南昌生态环境监测中心	AWA6228^{+}	JC-1318	93.8	93.5	94.0	AWA6223-F	FZ-0737
360100	2023	360103200058	象山路Ⅱ号	115.8897	28.6712	象山南路	1715	12	2	2	3	南昌市西湖区象山广场	0.1372	8	22	14	59	15	185	64.6	67.4	61.4	56.0	85.2	50.4	4.4	江西省南昌生态环境监测中心	AWA6228^{+}	JC-1265	93.8	93.9	94.0	AWA6223-F	FZ-0737
360100	2023	360112200031	学府大道Ⅰ号	115.7945	28.6536	学府大道	7414	44	6	2	2	南昌市红谷滩区南昌大学红角洲校区	0.4461	8	23	9	17	43	105	69.0	73.4	65.0	56.8	82.7	50.5	6.0	江西省南昌生态环境监测中心	AWA6228^{+}	JC-1319	93.8	93.7	94.0	AWA6223-F	FZ-0736
360100	2023	360102200037	沿江大道Ⅰ号	115.8892	28.7011	沿江北大道	6135	16	4	2	2	南昌市东湖区滨江宾馆	0.2454	8	22	10	2	5	969	73.4	76.2	72.8	67.0	82.3	57.5	3.6	江西省南昌生态环境监测中心	AWA6228^{+}	JC-1318	93.8	93.6	94.0	AWA6223-F	FZ-0737
360100	2023	360103200038	沿江大道Ⅱ号	115.8685	28.6710	沿江快速路	2926	18	6	2	1	南昌市西湖区海关大楼	0.117	8	23	10	47	18	1235	71.7	73.6	71.6	68.8	83.7	60.3	2.0	江西省南昌生态环境监测中心	AWA6228^{+}	JC-1317	93.8	93.7	94.0	AWA6223-F	FZ-0737
360100	2023	360103200040	沿江大道Ⅳ号	115.8421	28.6340	沿江快速路	4410	20	6	2	1	南昌市西湖区净业寺	0.1764	8	23	15	8	12	1920	68.7	71.2	67.8	66.4	81.2	63.7	1.9	江西省南昌生态环境监测中心	AWA6228^{+}	JC-1265	93.8	93.9	94.0	AWA6223-F	FZ-0737
360100	2023	360102200073	阳明路Ⅱ号	115.9084	28.6915	阳明东路	2597	39	6	2	2	南昌市东湖区贤士湖住宅小区	0.2078	8	22	10	33	19	964	68.4	69.2	66.6	62.8	92.8	59.7	2.8	江西省南昌生态环境监测中心	AWA6228^{+}	JC-1265	93.8	93.7	94.0	AWA6223-F	FZ-0737
360100	2023	360104200056	迎宾大道Ⅱ号	115.9028	28.6253	迎宾大道	5919	30	4	2	2	南昌市青云谱区江铃汽车股份公司	0.4735	8	23	9	58	42	1860	72.9	77.1	69.3	61.0	86.8	48.0	6.5	江西省南昌生态环境监测中心	AWA6228	JC-JS048-7	93.8	93.5	94.0	AWA6223-F	FZ-0735
360100	2023	360111200005	玉屏大街Ⅱ号	115.8178	28.7264	玉屏西大街	3587	16	4	2	2	南昌市青山湖区南昌凯马柴油机公司	0.195	8	21	15	20	52	152	59.0	62.0	54.4	49.0	83.7	41.4	5.0	江西省南昌生态环境监测中心	AWA6228^{+}	JC-1077	93.8	93.6	94.0	AWA6223-F	FZ-0736
360100	2023	360121200121	紫阳大道Ⅱ号	116.0140	28.6784	紫阳大道	6031	68	6	2	2	南昌市高新区蓝天学院瑶湖校区	0.3458	8	21	11	8	15	223	63.1	66.2	61.4	55.8	83.8	50.5	3.9	江西省南昌生态环境监测中心	AWA6228^{+}	JC-1317	93.8	94.0	94.0	AWA6223-F	FZ-0737
360100	2023	360121200123	艾溪湖南路Ⅰ	115.9932	28.6706	艾溪湖南路	4610	30	6	2	3	南昌市高新区中骏蓝湾香郡	0.2369	8	21	14	47	12	283	65.0	69.0	61.6	53.6	77.9	46.7	5.6	江西省南昌生态环境监测中心	AWA6228^{+}	JC-1076	93.8	93.6	94.0	AWA6223-F	FZ-0735

表3 2023年南昌市道路交通噪声昼间例行监测

行政区划代码	监测年度	点位编码	测点名称	测点经度	测点纬度	路段名称	路段长度（m）	道路总宽度（m）	机动车车道数	车道类别	道路等级	测点参照物	路段覆盖人口（万人）	月	日	时	分	大型车车流量	中小型车车流量	Leq	L10	L50	L90	最大值	最小值	标准差(SD)	监测站名	监测仪器型号	监测仪器编号	监测前校准值	监测后校准值	声校准器测量声压值	声校准仪器型号	声校准仪器编号
360100	2023	360121200124	艾溪湖南路Ⅱ	116.0180	28.6707	艾溪湖南路	2896	30	6	2	3	南昌市高新区小邹石村	0.1421	8	21	11	15	8	151	65.4	70.0	60.8	50.2	79.7	45.6	7.1	江西省南昌生态环境监测中心	AWA6228+	JC-1265	93.8	93.7	94.0	AWA6223-F	FZ-0737
360100	2023	360102200125	爱国路Ⅰ号	115.8884	28.6971	爱国路	3383	10	2	2	3	南昌市东湖区爱国路省军区	0.2706	8	22	10	17	3	165	69.8	72.8	64.4	57.0	91.6	49.6	6.2	江西省南昌生态环境监测中心	AWA6228+	JC-1317	93.8	93.9	94.0	AWA6223-F	FZ-0737
360100	2023	360112200126	白玉兰路Ⅰ号	115.7863	28.7100	白玉兰路	2915	20	4	2	3	南昌市新建区万科金域传奇	0.153	8	22	9	6	3	99	56.0	57.6	49.2	42.8	87.1	37.9	5.9	江西省南昌生态环境监测中心	AWA6228+	JC-1077	93.8	94.0	94.0	AWA6223-F	FZ-0736
360100	2023	360104200127	博学路Ⅰ号	115.9362	28.6298	博学路	1662	20	4	2	3	南昌市青云谱区博泰生命树小区	0.099	8	23	14	0	17	233	63.4	65.2	59.2	54.6	81.9	50.9	4.4	江西省南昌生态环境监测中心	AWA6228+	JC-1076	93.8	93.7	94.0	AWA6223-F	FZ-0735
360100	2023	360111200128	昌安路Ⅰ号	115.9713	28.6299	昌安路	3841	15	4	2	3	南昌市青山湖区华奥印务有限公司	0.2268	8	21	16	17	7	133	64.1	68.2	56.4	51.8	80.1	49.9	6.3	江西省南昌生态环境监测中心	AWA6228+	JC-1076	93.8	93.7	94.0	AWA6223-F	FZ-0735
360100	2023	360112200129	城运大道Ⅰ号	115.8104	28.6032	城运大道	8480	40	6	2	3	南昌市红谷滩区南昌光年装饰有限公司	0.1808	8	21	15	34	0	267	65.0	68.2	60.0	48.4	84.4	43.3	7.5	江西省南昌生态环境监测中心	AWA6228+	JC-1318	93.8	93.9	94.0	AWA6223-F	FZ-0737
360100	2023	360121200130	创新一路Ⅰ号	115.9979	28.6989	创新一路	4743	10	2	2	3	南昌市高新区千禧同业	0.2808	8	21	10	35	3	136	64.2	66.8	60.8	52.8	86.4	46.1	5.5	江西省南昌生态环境监测中心	AWA6228+	JC-1315	93.8	93.6	94.0	AWA6223-F	FZ-0735
360100	2023	360111200131	东泰大道Ⅰ号	115.9696	28.6382	东泰大道	6157	18	4	2	3	南昌市青山湖区江苏皮尔萨管业	0.4161	8	21	15	51	6	187	66.9	69.6	64.4	58.3	89.2	51.5	4.6	江西省南昌生态环境监测中心	AWA6228	JC-JS030	93.8	93.9	94.0	AWA6223-F	FZ-0735
360100	2023	360102200132	二七北路Ⅰ号	115.9135	28.6805	二七北路	3021	16	4	2	3	南昌市东湖区东来顺饭庄对面	0.2417	8	22	11	17	8	267	66.6	70.2	63.6	57.8	85.3	50.4	4.6	江西省南昌生态环境监测中心	AWA6228+	JC-1317	93.8	93.9	94.0	AWA6223-F	FZ-0737
360100	2023	360102200133	福州路Ⅰ号	115.9085	28.6866	福州路	1539	15	4	2	3	南昌市东湖区金昌利大厦对面	0.1231	8	22	11	8	3	148	62.9	65.2	60.6	56.6	83.4	53.4	3.5	江西省南昌生态环境监测中心	AWA6228+	JC-1318	93.8	93.9	94.0	AWA6223-F	FZ-0737
360100	2023	360111200134	富大有路Ⅰ号	115.9503	28.7323	富大有路	6324	30	6	2	3	南昌市青山湖区万科金域滨江	0.133	8	21	11	32	26	491	68.8	72.9	62.6	58.6	86.9	53.3	5.8	江西省南昌生态环境监测中心	AWA6228	JC-JS030	93.8	93.6	94.0	AWA6223-F	FZ-0735
360100	2023	360121200135	富大有路Ⅱ号	115.9963	28.7248	富大有路	6741	40	6	2	3	南昌市高新区白云驾校	0.0708	8	21	10	22	56	873	69.6	72.6	67.4	61.8	83.0	56.0	4.2	江西省南昌生态环境监测中心	AWA6228+	JC-1076	93.8	93.6	94.0	AWA6223-F	FZ-0735
360100	2023	360111200136	高新七路Ⅰ号	115.9714	28.7039	高新七路	2180	15	4	2	3	南昌市青山湖区南昌外国语高新学校	0.1744	8	22	14	0	15	210	64.3	68.2	61.2	52.8	79.7	47.1	5.8	江西省南昌生态环境监测中心	AWA6228+	JC-1076	93.8	93.6	94.0	AWA6223-F	FZ-0735
360100	2023	360112200137	工业大道Ⅰ号	115.7854	28.6991	工业大道	2977	40	6	2	2	南昌市新建区南昌顺发机动车检测有限公司	0.1902	8	22	9	20	35	198	66.9	70.4	64.0	55.0	87.8	48.6	5.9	江西省南昌生态环境监测中心	AWA6228+	JC-1319	93.8	93.7	94.0	AWA6223-F	FZ-0736
360100	2023	360111200138	海棠南路Ⅰ号	115.8086	28.7311	海棠南路	2451	28	4	2	3	南昌市青山湖区江西财大麦庐校区	0.1481	8	21	14	35	12	48	64.5	66.8	56.8	51.0	90.3	48.2	6.2	江西省南昌生态环境监测中心	AWA6228+	JC-1319	93.8	93.6	94.0	AWA6223-F	FZ-0736
360100	2023	360112200139	黄家湖东路Ⅰ	115.8239	28.7106	黄家湖东路	2355	40	6	2	2	南昌市新建区奥克斯盛世华庭南门	0.1395	8	21	15	38	17	189	65.0	67.6	62.4	54.2	85.6	45.0	5.3	江西省南昌生态环境监测中心	AWA6228+	JC-1319	93.8	93.9	94.0	AWA6223-F	FZ-0736
360100	2023	360111200140	黄家湖西路Ⅰ	115.8045	28.7162	黄家湖西路	4188	40	6	2	2	南昌市青山湖区汉港凯旋城	0.2062	8	22	11	0	14	107	57.6	56.8	49.4	42.2	84.2	35.3	5.8	江西省南昌生态环境监测中心	AWA6228+	JC-1077	93.8	93.9	94.0	AWA6223-F	FZ-0736
360100	2023	360111200141	火炬二路Ⅰ号	115.9592	28.6973	火炬二路	2765	10	2	2	3	南昌市青山湖区大唐烧烤	0.2212	8	24	9	44	8	187	64.3	67.2	61.0	55.4	91.8	50.1	4.5	江西省南昌生态环境监测中心	AWA6228+	JC-1076	93.8	93.6	94.0	AWA6223-F	FZ-0735
360100	2023	360111200142	火炬三路Ⅰ号	115.9665	28.7048	火炬三路	2109	10	4	2	3	南昌市青山湖区汉庭酒店	0.1687	8	22	14	2	7	105	63.7	67.7	59.8	53.4	87.4	49.8	5.8	江西省南昌生态环境监测中心	AWA6228	JC-JS030	93.8	93.6	94.0	AWA6223-F	FZ-0735
360100	2023	360111200143	火炬五路Ⅰ号	115.9726	28.7184	火炬五路	2914	18	4	2	3	南昌市青山湖区百利精密刀具（南昌）有限公司	0.2331	8	21	11	23	5	76	66.9	67.6	59.0	51.8	103.2	30.0	6.0	江西省南昌生态环境监测中心	AWA6228+	JC-1315	93.8	94.1	94.0	AWA6223-F	FZ-0735
360100	2023	360111200144	金港路Ⅰ号	115.8820	28.7688	金港路	4441	24	4	2	3	南昌市青山湖区江西华盛钢铁实业有限公司	0.2278	8	21	10	8	74	167	73.0	75.8	66.6	55.6	94.0	51.5	7.6	江西省南昌生态环境监测中心	AWA6228+	JC-1319	93.8	93.7	94.0	AWA6223-F	FZ-0736
360100	2023	360111200145	金山大道Ⅰ号	115.9168	28.7736	金山大道	8983	50	6	2	2	南昌市青山湖区洪城警苑小区	0.0961	8	21	9	40	35	15	69.5	72.6	64.0	60.8	87.4	58.6	4.7	江西省南昌生态环境监测中心	AWA6228+	JC-1316	93.8	93.5	94.0	AWA6223-F	FZ-0736
360100	2023	360111200146	京安路Ⅰ号	115.9611	28.6715	京安路	1525	21	4	2	3	南昌市青山湖区水之韵养生馆	0.122	8	21	14	45	7	234	65.2	68.4	62.2	55.0	86.3	49.0	5.1	江西省南昌生态环境监测中心	AWA6228+	JC-1315	93.8	93.7	94.0	AWA6223-F	FZ-0735
360100	2023	360111200147	经开大道Ⅰ号	115.8766	28.7721	经开大道	7598	34	6	2	2	南昌市青山湖区南昌志航无缝钢管球墨铸铁管公司	0.302	8	21	10	3	18	66	73.0	74.6	63.4	54.0	94.7	48.5	7.9	江西省南昌生态环境监测中心	AWA6228+	JC-1077	93.8	94.0	94.0	AWA6223-F	FZ-0736
360100	2023	360112200148	景德镇街Ⅰ号	115.7911	28.6153	景德镇街	3551	27	6	2	3	南昌市红谷滩区腾龙学校	0.142	8	21	15	21	2	86	58.0	62.6	49.8	44.0	76.8	39.2	7.1	江西省南昌生态环境监测中心	AWA6228+	JC-1265	93.8	93.8	94.0	AWA6223-F	FZ-0737
360100	2023	360112200149	九龙大道Ⅰ号	115.7912	28.6053	九龙大道	7583	75	12	2	2	南昌市红谷滩区绿地国际博览中心	0.2443	8	21	16	8	6	292	67.8	72.6	61.4	55.2	80.3	49.4	6.4	江西省南昌生态环境监测中心	AWA6228+	JC-1265	93.8	93.9	94.0	AWA6223-F	FZ-0737
360100	2023	360111200150	梅林大道Ⅰ号	115.8741	28.7606	梅林大道	2919	28	4	2	3	南昌市青山湖区海立电器	0.2335	8	21	10	29	20	18	67.8	71.0	62.4	58.6	87.9	56.7	4.8	江西省南昌生态环境监测中心	AWA6228+	JC-1316	93.8	93.9	94.0	AWA6223-F	FZ-0736
360100	2023	360112200151	梅岭大道Ⅰ号	115.7456	28.6829	梅岭大道	3956	34	6	2	2	南昌市新建区湾里江西中医大学科技学院	0.1727	8	23	11	28	2	53	63.4	68.0	56.6	47.8	77.1	40.1	7.5	江西省南昌生态环境监测中心	AWA6228+	JC-1319	93.8	94.2	94.0	AWA6223-F	FZ-0736
360100	2023	360111200152	民营大道Ⅰ号	115.9500	28.7161	民营大道	2084	30	4	2	3	南昌市青山湖区星辉村安置房	0.1667	8	22	11	40	16	169	64.4	67.2	60.4	53.5	92.4	48.5	6.0	江西省南昌生态环境监测中心	AWA6228	JC-JS030	93.8	93.5	94.0	AWA6223-F	FZ-0735
360100	2023	360111200153	民营大道Ⅱ号	115.9498	28.7019	民营大道	1991	30	4	2	3	南昌市青山湖区车漆世界钣喷中心	0.1593	8	22	11	31	5	103	62.1	64.8	59.4	54.6	89.3	52.3	3.9	江西省南昌生态环境监测中心	AWA6228+	JC-1076	93.8	93.5	94.0	AWA6223-F	FZ-0735
360100	2023	360112200154	明矾路Ⅰ号	115.8023	28.7089	明矾路	2054	26	6	2	3	南昌市新建区城西市容环境服务站	0.1643	8	21	16	41	12	85	62.6	62.0	55.4	50.0	90.3	45.7	4.7	江西省南昌生态环境监测中心	AWA6228+	JC-1319	93.8	93.7	94.0	AWA6223-F	FZ-0736
360100	2023	360112200155	南龙蟠街Ⅰ号	115.7901	28.5827	南龙蟠街	2481	28	6	2	3	南昌市红谷滩区九龙湖南地铁站东	0.0992	8	21	16	23	3	184	60.7	63.6	58.4	53.8	77.6	49.8	3.8	江西省南昌生态环境监测中心	AWA6228+	JC-1318	93.8	93.4	94.0	AWA6223-F	FZ-0737
360100	2023	360111200156	青山湖大道Ⅰ	115.9453	28.7072	青山湖大道	5117	29	6	2	2	南昌市青山湖区二七王食府	0.4094	8	22	14	36	8	105	62.6	66.4	59.8	55.8	77.2	52.8	4.0	江西省南昌生态环境监测中心	AWA6228+	JC-1076	93.8	94.0	94.0	AWA6223-F	FZ-0735
360100	2023	360111200157	青山湖大道Ⅱ	115.9445	28.6683	青山湖大道	3930	29	6	2	2	南昌市青山湖区南铁世纪花园	0.3144	8	22	15	29	54	634	64.8	68.4	62.4	55.0	83.9	45.5	5.1	江西省南昌生态环境监测中心	AWA6228+	JC-1076	93.8	93.7	94.0	AWA6223-F	FZ-0735
360100	2023	360104200158	墅溪路Ⅰ号	115.9286	28.6474	墅溪路	2715	26	6	2	3	南昌市青云谱区保利和光小区入口	0.2172	8	22	16	49	8	231	68.4	71.1	67.1	63.0	84.8	56.0	4.3	江西省南昌生态环境监测中心	AWA6228	JC-JS030	93.8	94.0	94.0	AWA6223-F	FZ-0735

表3　2023年南昌市道路交通噪声昼间例行监测

行政区划代码	监测年度	点位编码	测点名称	测点经度	测点纬度	路段名称	路段长度（m）	道路总宽度（m）	机动车车道	车道类别	道路等级	测点参照物	路段覆盖人口（万人）	月	日	时	分	大型车车流量	中小型车车流量	Leq	L10	L50	L90	最大值	最小值	标准差（SD）	监测站名	监测仪器型号	监测仪器编号	监测前校准值	监测后校准值	声校准器测量声压值	声校准仪器型号	声校准仪器编号
360100	2022	360112200159	双马石路Ⅰ号	115.7266	28.7018	双马石路	1861	16	4	2	3	南昌市新建区湾里天安财险	0.0981	8	23	10	55	5	76	70.2	74.0	67.2	58.0	87.5	49.3	5.9	江西省南昌生态环境监测中心	AWA6228+	JC-1319	93.8	93.7	94.0	AWA6223-F	FZ-0736
360100	2023	360112200160	天宁路Ⅰ号	115.7291	28.6945	天宁路	3808	26	4	2	3	南昌市新建区湾里金佳·云舒花城	0.184	8	23	11	20	8	120	51.8	57.0	49.6	41.8	78.9	32.4	6.0	江西省南昌生态环境监测中心	AWA6228+	JC-1077	93.8	93.6	94.0	AWA6223-F	FZ-0736
360100	2022	360112200161	文化大道Ⅰ号	115.8196	28.7002	文化大道	3577	34	4	2	2	南昌市新建区米罗小区南门	0.2106	8	21	16	25	24	341	51.2	55.6	49.8	42.6	80.4	34.2	5.3	江西省南昌生态环境监测中心	AWA6228+	JC-1077	93.8	93.7	94.0	AWA6223-F	FZ-0736
360100	2023	360112200162	武功山大道Ⅰ	115.7358	28.6359	武功山大道	9712	55	6	2	2	南昌市新建区联富花园A期7栋旁	0.561	8	21	14	42	46	402	66.6	71.4	61.2	51.4	83.6	43.7	7.2	江西省南昌生态环境监测中心	AWA6228+	JC-1265	93.8	93.9	94.0	AWA6223-F	FZ-0737
360100	2022	360104200163	新溪桥路Ⅰ号	115.9175	28.6397	新溪桥路	2238	24	6	2	3	南昌市青云谱区旺中旺生活超市洪都店	0.179	8	23	10	11	6	28	63.2	63.7	59.4	57.4	85.4	55.3	3.7	江西省南昌生态环境监测中心	AWA6228	JC-JS030	93.8	93.3	94.0	AWA6223-F	FZ-0735
360100	2023	360112200164	兴国路Ⅰ号	115.8037	28.6940	兴国路	4086	16	4	2	3	南昌市新建区警犬基地动物医院	0.2789	8	22	9	29	33	76	71.2	70.8	65.2	61.0	97.4	53.8	4.6	江西省南昌生态环境监测中心	AWA6228+	JC-1316	93.8	93.9	94.0	AWA6223-F	FZ-0736
360100	2022	360111200165	秀先路Ⅰ号	115.8977	28.7844	秀先路	2004	40	4	2	3	南昌市青山湖区南昌攀藤科技有限公司	0.048	8	21	9	12	7	60	57.7	62.0	51.8	46.6	79.3	44.1	5.7	江西省南昌生态环境监测中心	AWA6228+	JC-1077	93.8	93.7	94.0	AWA6223-F	FZ-0736
360100	2023	360121200166	瑶湖西大道Ⅰ	116.0335	28.6830	瑶湖西大道	3570	25	6	2	3	南昌市高新区昌东镇黄合高新现代产业综合城	0.0902	8	21	11	0	32	231	67.8	71.8	63.8	53.2	82.0	47.2	6.8	江西省南昌生态环境监测中心	AWA6228+	JC-1318	93.8	94.0	94.0	AWA6223-F	FZ-0737
360100	2022	360121200167	瑶湖西大道Ⅱ	116.0437	28.7138	瑶湖西大道	4077	25	6	2	3	南昌市高新区昌东镇君江半岛	0.0956	8	21	9	45	28	207	63.5	66.8	59.0	51.2	85.7	46.2	5.8	江西省南昌生态环境监测中心	AWA6228+	JC-1318	93.8	93.7	94.0	AWA6223-F	FZ-0737
360100	2023	360121200168	瑶湖西一路Ⅰ	116.0232	28.6880	瑶湖西一路	2329	13	4	2	3	南昌市高新区昌东镇南昌工程学院	0.1863	8	21	10	21	4	88	67.7	71.4	61.8	54.4	87.6	49.8	6.3	江西省南昌生态环境监测中心	AWA6228+	JC-1265	93.8	93.6	94.0	AWA6223-F	FZ-0737
360100	2022	360111200169	英雄大道Ⅰ号	115.8694	28.7818	英雄大道	6005	24	6	2	3	南昌市青山湖区五联花园小区	0.2726	8	21	9	24	36	115	67.2	70.6	59.2	54.6	86.8	50.8	6.2	江西省南昌生态环境监测中心	AWA6228+	JC-1319	93.8	93.9	94.0	AWA6223-F	FZ-0736
360100	2023	360112200170	永强路Ⅰ号	115.7290	28.6404	永强路	4096	18	4	2	3	南昌市新建区太阳岛文具厂对面	0.3277	8	21	14	28	1	37	60.5	61.8	54.6	52.2	82.5	50.2	4.4	江西省南昌生态环境监测中心	AWA6228+	JC-1318	93.8	93.5	94.0	AWA6223-F	FZ-0737
360100	2022	360112200171	长堎大道Ⅰ号	115.7965	28.6894	长堎大道	3296	26	6	2	3	南昌市新建区江西新一建筑工程公司门口	0.195	8	22	10	13	5	350	63.2	65.0	62.0	57.8	82.4	51.7	3.1	江西省南昌生态环境监测中心	AWA6228+	JC-1316	93.8	93.7	94.0	AWA6223-F	FZ-0736
360100	2023	360112200172	长麦路Ⅰ号	115.8135	28.7041	长麦路	3100	24	6	2	3	南昌市新建区南昌新建一中	0.1991	8	22	10	50	5	130	62.4	65.0	58.2	52.6	86.6	48.3	4.9	江西省南昌生态环境监测中心	AWA6228+	JC-1316	93.8	93.7	94.0	AWA6223-F	FZ-0736
360100	2022	360112200173	长麦南路Ⅰ号	115.8145	28.6850	长麦南路	1581	24	6	2	3	南昌市新建区幸福里小区12栋旁	0.1265	8	22	10	20	8	93	60.2	62.8	57.4	51.2	83.3	43.9	4.6	江西省南昌生态环境监测中心	AWA6228+	JC-1077	93.8	93.7	94.0	AWA6223-F	FZ-0736
360100	2023	360112200174	长征西路Ⅰ号	115.8169	28.6829	长征西路	4123	15	6	2	3	南昌市新建区长征西路馨居雅苑对面	0.2202	8	23	11	39	37	82	64.9	67.0	63.2	59.4	87.2	55.6	3.1	江西省南昌生态环境监测中心	AWA6228+	JC-1316	93.8	93.9	94.0	AWA6223-F	FZ-0736
360100	2022	360112200175	招贤大道Ⅰ号	115.7339	28.7092	招贤大道	4292	38	6	2	2	南昌市新建区湾里COCO公馆	0.3019	8	23	10	45	12	170	55.3	58.4	51.4	42.0	81.7	34.0	6.3	江西省南昌生态环境监测中心	AWA6228+	JC-1077	93.8	93.7	94.0	AWA6223-F	FZ-0736
360100	2023	360104200176	朱桥东路Ⅰ号	115.9336	28.6108	朱桥东路	3151	30	4	2	3	南昌市青云谱区深圳农产品中心批发市场冻品区十号门	0.2141	8	23	11	8	60	110	62.6	65.0	58.9	55.3	83.9	46.7	4.8	江西省南昌生态环境监测中心	AWA6228	JC-JS048-7	93.8	93.6	94.0	AWA6223-F	FZ-0735
360121	2022	360121200001	三幼汇仁	115.9045	28.5712	工业一路	2552	12	4	2	3	南昌市南昌县三幼汇仁园区	0.16	2	23	14	3	10	167	66.5	69.2	60.0	53.2	85.7	49.5	6.1	江西省南昌生态环境监测中心	AWA6228+	JC-1264	93.8	93.7	94.0	AWA6021A	FZ-0736
360121	2023	360121200002	金沙幼儿园	115.8642	28.5667	金沙三路	7676	22	4	2	2	南昌市南昌县卓尔金沙幼儿园	0.19	2	14	14	1	18	293	72.2	74.8	68.2	59.4	92.0	49.9	6.1	江西省南昌生态环境监测中心	AWA6228+	JC-1076	93.8	93.9	94.0	AWA6021A	FZ-0735
360121	2022	360121200003	泰豪动漫学院	115.8743	28.5761	金沙二路	3360	16	4	2	2	南昌市南昌县泰豪动漫学院	0.29	2	14	14	34	10	408	67.1	70.2	65.2	59.0	80.6	52.7	4.4	江西省南昌生态环境监测中心	AWA6228+	JC-1076	93.8	93.8	94.0	AWA6021A	FZ-0735
360121	2023	360121200004	无端道医堂	115.8685	28.5830	东莲路	6117	26	6	2	2	南昌市南昌县新力幸福时光无端道医堂	0.17	2	14	17	3	2	255	66.8	71.0	62.8	55.2	86.0	47.3	6.0	江西省南昌生态环境监测中心	AWA6228+	JC-1077	93.8	93.8	94.0	AWA6021A	FZ-0737
360121	2022	360121200005	智慧树幼儿园	115.9221	28.5786	康莲路	1270	30	4	2	3	南昌市南昌县智慧树幼儿园	0.08	2	23	15	2	1	75	57.5	60.4	53.8	49.8	75.4	46.1	4.2	江西省南昌生态环境监测中心	AWA6228+	JC-1264	93.8	93.6	94.0	AWA6021A	FZ-0736
360121	2023	360121200006	百威雪津	115.8829	28.5615	金沙大道	18067	29	6	2	2	南昌市南昌县百威雪津（南昌）啤酒有限公司	0.35	2	14	15	44	15	668	71.3	74.4	69.8	60.8	93.2	50.8	5.3	江西省南昌生态环境监测中心	AWA6228+	JC-1076	93.8	93.8	94.0	AWA6021A	FZ-0735
360121	2022	360121200007	煌盛中央公园	115.9092	28.5687	迎宾中大道	6648	80	6	2	2	南昌市南昌县煌盛中央公园东门欧润油品	0.54	2	23	10	22	64	877	64.9	66.6	62.2	58.2	84.5	52.8	3.7	江西省南昌生态环境监测中心	AWA6228+	JC-1264	93.8	93.7	94.0	AWA6021A	FZ-0736
360121	2023	360121200008	绵竹幸福里	115.8601	28.6051	八月湖路	4305	24	8	2	2	南昌市南昌县绵竹幸福里七彩色地产	0.24	2	14	15	5	17	63	66.4	69.4	63.0	54.4	87.3	48.6	5.6	江西省南昌生态环境监测中心	AWA6228+	JC-1077	93.8	94.0	94.0	AWA6021A	FZ-0737
360121	2022	360121200009	景城名郡	115.8792	28.5886	芳草路	1979	12	4	2	3	南昌市南昌县景城名郡我爱我家	0.21	2	14	16	23	12	58	66.1	67.8	64.0	61.4	88.5	57.4	2.8	江西省南昌生态环境监测中心	AWA6228+	JC-1077	93.8	94.1	94.0	AWA6021A	FZ-0737
360121	2023	360121200010	棕榈学府	115.9140	28.5538	莲安南路	600	28	4	2	3	南昌市南昌县棕榈学府东新芝教	0.16	2	23	16	47	13	112	61.9	63.6	57.6	53.8	88.1	49.7	4.1	江西省南昌生态环境监测中心	AWA6228+	JC-1264	93.8	93.9	94.0	AWA6021A	FZ-0736
360121	2022	360121200011	五羊本田	115.9303	28.5792	南莲路	5335	46	6	2	2	南昌市南昌县鹿鼎爱丁堡五羊本田	0.48	2	23	15	30	66	411	69.4	71.4	68.0	61.6	100.5	53.0	4.0	江西省南昌生态环境监测中心	AWA6228+	JC-1264	93.8	93.9	94.0	AWA6021A	FZ-0736
360121	2023	360121200012	盐业包装	115.8881	28.5526	富山一路	4115	10	2	2	3	南昌市南昌县江西盐业包装有限公司马路对面	0.08	2	14	16	16	6	104	66.3	68.8	57.4	51.0	91.6	47.5	6.8	江西省南昌生态环境监测中心	AWA6228+	JC-1076	93.8	93.8	94.0	AWA6021A	FZ-0735
360121	2022	360121200013	中国民生银行	115.9274	28.5525	澄湖西路	3400	40	4	2	2	南昌市南昌县中国民生银行澄湖西路支行	0.58	2	14	15	14	5	293	69.2	73.0	66.8	58.6	82.8	51.0	5.4	江西省南昌生态环境监测中心	AWA6228+	JC-1265	93.8	93.9	94.0	AWA6021A	FZ-0736
360121	2023	360121200014	味思诺蛋糕	115.9375	28.5279	安宝大道	1100	40	6	2	3	南昌市南昌县银河城味思诺蛋糕	0.21	2	14	16	56	21	249	68.1	71.6	65.8	58.6	84.8	51.6	4.9	江西省南昌生态环境监测中心	AWA6228+	JC-1265	93.8	93.8	94.0	AWA6021A	FZ-0736
360121	2022	360121200015	居住主题公园	115.8838	28.5959	海嘉路	1561	12	4	2	3	南昌市南昌县南昌居住主题公园19栋西面	0.26	2	14	15	40	0	60	61.6	63.8	55.0	51.0	93.3	46.7	5.1	江西省南昌生态环境监测中心	AWA6228+	JC-1077	93.8	93.8	94.0	AWA6021A	FZ-0737
360121	2023	360121200016	新力象湖湾	115.8362	28.5944	东岳大道	4842	16	8	2	2	南昌市南昌县新力象湖湾南门京东家电	0.20	2	14	14	6	0	260	63.8	67.6	59.6	51.2	82.9	46.6	6.1	江西省南昌生态环境监测中心	AWA6228+	JC-1077	93.8	93.5	94.0	AWA6021A	FZ-0737
360121	2022	360121200017	梦里水乡	115.9198	28.5598	澄湖北路	2700	68	8	2	1	南昌市南昌县梦里水乡北门中国太平洋保险	0.24	2	23	16	7	11	668	64.5	68.0	62.8	57.2	75.8	50.6	4.1	江西省南昌生态环境监测中心	AWA6228+	JC-1264	93.8	93.7	94.0	AWA6021A	FZ-0736

表3　2023年南昌市道路交通噪声昼间例行监测

行政区划代码	监测年度	点位编码	测点名称	测点经度	测点纬度	路段名称	路段长度（m）	道路总宽度（m）	机动车车道数	车道类别	道路等级	测点参照物	路段覆盖人口（万人）	月	日	时	分	大型车车流量	中小型车车流量	Leq	L10	L50	L90	最大值	最小值	标准差（SD）	监测站名	监测仪器型号	监测仪器编号	监测前校准值	监测后校准值	声校准器测量声压值	声校准仪器型号	声校准仪器编号
360121	2023	360121200018	东新乡政府	115.8424	28.6049	东祥路	5020	24	6	2	2	南昌市南昌县东新乡政府东面	0.16	2	14	14	35	0	180	64.6	68.0	62.0	55.2	87.5	49.7	4.8	江西省南昌生态环境监测中心	AWA6228+	JC-1077	93.8	93.8	94.0	AWA6021A	FZ-0737
360121	2023	360121200019	力高君御华府	115.9280	28.5407	莲富路	1921	14	4	2	2	南昌市南昌县力高君御华府圣达文化艺术培训学校	0.12	2	14	15	47	0	169	64.2	67.4	61.8	57.2	85.8	53.7	3.8	江西省南昌生态环境监测中心	AWA6228+	JC-1265	93.8	93.8	94.0	AWA6021A	FZ-0736
360121	2023	360121200020	三志物流	115.8665	28.5445	富山大道	9919	20	6	2	2	南昌市南昌县富山大道290号南昌三志物流有限公司	0.21	2	14	16	52	36	703	70.4	73.2	69.4	63.8	90.9	57.3	3.6	江西省南昌生态环境监测中心	AWA6228+	JC-1076	93.8	93.9	94.0	AWA6021A	FZ-0735
360121	2023	360121200021	迪启博幼儿园	115.9395	28.5429	五一路	1253	10	2	2	2	南昌市南昌县迪启博幼儿园	0.08	2	14	16	27	0	89	64.2	66.2	60.6	57.0	90.1	52.9	3.9	江西省南昌生态环境监测中心	AWA6228+	JC-1265	93.8	93.7	94.0	AWA6021A	FZ-0736
360121	2023	360121200022	新力银湖湾	115.8890	28.5732	汇仁大道	6043	28	6	2	2	南昌市南昌县新力银湖湾中国电信	0.25	2	14	15	8	24	659	74.1	77.4	72.6	63.6	85.1	51.9	5.5	江西省南昌生态环境监测中心	AWA6228+	JC-1076	93.8	93.8	94.0	AWA6021A	FZ-0735
360121	2023	360121200023	烨达环保	115.8896	28.5388	富山二路	4154	9	2	2	3	南昌市南昌县江苏烨达环保驻江西生产基地	0.05	2	14	14	35	5	53	64.5	67.2	60.0	57.6	83.0	55.2	4.0	江西省南昌生态环境监测中心	AWA6228+	JC-1265	93.8	93.7	94.0	AWA6021A	FZ-0736
360121	2023	360121200024	宝江钢材	115.8862	28.5251	富山五路（汽车大道）	4972	30	6	2	2	南昌市南昌县宝江钢材股份有限公司	0.15	2	14	14	2	32	375	68.9	72.8	61.8	55.6	88.6	51.4	6.5	江西省南昌生态环境监测中心	AWA6228+	JC-1265	93.8	93.8	94.0	AWA6021A	FZ-0736
360123	2023	360123200001	育苗幼儿园	115.5417	28.8475	北门路	735	10	1	1	3	南昌市安义县育苗幼儿园	0.5	4	17	17	6	0	30	63.2	65.8	60.2	56.6	86.2	51.4	3.7	江西省南昌生态环境监测中心	AWA6228+	JC-1265	93.8	93.6	94.0	AWA6223-F	FZ-0735
360123	2023	360123200002	农税征收局	115.5437	28.8452	解放路	1233	20	2	2	3	南昌市安义县农业税征收管理局	0.6	4	17	16	43	1	42	65.3	66.6	60.4	56.0	91.5	52.1	4.3	江西省南昌生态环境监测中心	AWA6228+	JC-1264	93.8	93.6	94.0	AWA6223-F	FZ-0735
360123	2023	360123200003	前进村卫生所	115.5490	28.8427	东门路	1300	25	2	2	2	南昌市安义县龙津镇前进村卫生所	0.8	4	17	16	37	0	72	66.6	68.6	64.4	60.6	87.3	56.2	3.4	江西省南昌生态环境监测中心	AWA6228+	JC-1265	93.8	93.9	94.0	AWA6223-F	FZ-0735
360123	2023	360123200004	鑫辉鞋业	115.5490	28.8426	人民路	1000	22	2	2	3	南昌市安义县鑫辉鞋业	0.9	4	17	16	38	1	63	64.6	66.6	60.8	57.4	85.5	52.7	3.9	江西省南昌生态环境监测中心	AWA6228+	JC-1076	93.8	93.6	94.0	AWA6223-F	FZ-0735
360123	2023	360123200005	金凤花园	115.5607	28.8465	延雄路	1000	24	4	2	2	南昌市安义县金凤花园管理处	0.9	4	17	15	34	0	54	61.7	64.8	55.6	48.8	80.3	45.7	6.2	江西省南昌生态环境监测中心	AWA6228+	JC-1264	93.8	94.0	94.0	AWA6223-F	FZ-0735
360123	2023	360123200006	蓝调咖啡	115.5678	28.8474	达瑞路	1000	20	4	2	3	南昌市安义县蓝调咖啡	0.5	4	17	15	31	1	80	60.7	64.2	56.0	48.8	77.5	42.7	5.9	江西省南昌生态环境监测中心	AWA6228+	JC-1076	93.8	93.5	94.0	AWA6223-F	FZ-0735
360123	2023	360123200007	香榭大道	115.5733	28.8496	学府大道	1050	24	4	2	2	南昌市安义县香榭大道二期	0.3	4	17	15	28	1	51	63.8	66.8	56.0	48.8	86.3	42.3	7.1	江西省南昌生态环境监测中心	AWA6228+	JC-1265	93.8	93.7	94.0	AWA6223-F	FZ-0735
360123	2023	360123200008	伟星实业	115.5819	28.8544	凤凰大道	2064	32	4	2	2	南昌市安义县江西伟星实业	0.1	4	17	15	1	0	77	64.7	68.6	56.0	48.4	87.5	44.1	7.5	江西省南昌生态环境监测中心	AWA6228+	JC-1076	93.8	93.7	94.0	AWA6223-F	FZ-0735
360123	2023	360123200009	德旺不锈钢	115.5867	28.8459	引凤路	1575	25	4	2	2	南昌市安义县江西安邦德旺不锈钢	0.2	4	17	14	58	1	35	62.6	64.0	58.8	53.8	84.5	52.2	4.4	江西省南昌生态环境监测中心	AWA6228+	JC-1265	93.8	93.4	94.0	AWA6223-F	FZ-0735
360123	2023	360123200010	兰珠实业	115.5951	28.8589	孙虑成东路	2298	32	2	2	2	南昌市安义县南昌市兰珠实业	0.1	4	17	14	10	11	50	65.5	67.6	58.4	54.2	89.3	52.7	5.4	江西省南昌生态环境监测中心	AWA6228+	JC-1265	93.8	93.5	94.0	AWA6223-F	FZ-0735
360123	2023	360123200011	勇居康酒店	115.6061	28.8612	凤凰东路	2104	40	2	2	2	南昌市安义县勇居康商务酒店	0.1	4	17	11	23	11	65	63.8	67.8	56.8	51.8	86.0	49.5	6.1	江西省南昌生态环境监测中心	AWA6228+	JC-1265	93.8	93.5	94.0	AWA6223-F	FZ-0735
360123	2023	360123200012	新凤玉石	115.5886	28.8650	创业大道	2251	36	4	2	2	南昌市安义县江西新凤微晶玉石公司	0.1	4	17	14	14	7	47	66.7	69.4	64.4	63.0	81.8	57.6	2.7	江西省南昌生态环境监测中心	AWA6228+	JC-1076	93.8	93.7	94.0	AWA6223-F	FZ-0735
360123	2023	360123200013	荣凯铝业	115.5964	28.8620	锦绣大道	2768	40	4	2	3	南昌市安义县荣凯铝业	0.1	4	17	11	30	25	115	69.0	71.8	63.4	57.0	97.9	53.6	5.6	江西省南昌生态环境监测中心	AWA6228+	JC-1264	93.8	93.3	94.0	AWA6223-F	FZ-0735
360123	2023	360123200014	和祥铝业	115.6028	28.8585	起秀路	1700	16	4	2	2	南昌市安义县江西和祥铝业	0.1	4	17	11	29	39	5	61.7	63.4	54.4	52.4	84.5	50.4	4.8	江西省南昌生态环境监测中心	AWA6228+	JC-1076	93.8	93.7	94.0	AWA6223-F	FZ-0735
360123	2023	360123200015	安义县华荟苑	115.5511	28.8498	建设路	1409	25	2	2	2	南昌市安义县华荟苑	0.5	4	17	16	5	0	66	61.9	65.8	57.6	50.0	79.8	44.7	5.8	江西省南昌生态环境监测中心	AWA6228+	JC-1264	93.8	93.6	94.0	AWA6223-F	FZ-0735
360123	2023	360123200016	安义县阳湖村	115.5538	28.8466	向阳路	2743	24	4	2	2	南昌市安义县龙津镇阳湖村前房文化活动中心	0.9	4	17	16	3	0	36	63.0	65.4	56.0	48.8	89.9	40.9	6.5	江西省南昌生态环境监测中心	AWA6228+	JC-1076	93.8	93.5	94.0	AWA6223-F	FZ-0735
360123	2023	360123200017	安义县清和园	115.5765	28.8466	前进路	4125	32	4	2	2	南昌市安义县清和园	0.6	4	17	15	4	1	100	64.7	67.8	59.4	52.0	83.3	45.8	6.0	江西省南昌生态环境监测中心	AWA6228+	JC-1264	93.8	93.5	94.0	AWA6223-F	FZ-0735
360123	2023	360123200018	安义中学	115.5549	28.8420	文峰路	1639	32	6	2	2	南昌市安义县安义中学	0.9	4	17	16	0	6	190	66.4	69.8	64.0	58.2	87.0	50.0	4.4	江西省南昌生态环境监测中心	AWA6228+	JC-1265	93.8	93.7	94.0	AWA6223-F	FZ-0735
360123	2023	360123200019	安义县滤城园	115.5908	28.8454	迎宾大道	2000	55	2	2	3	南昌市安义县滤城园农家饭庄	0.2	4	17	14	32	22	236	70.0	73.4	66.0	57.2	90.8	52.1	6.1	江西省南昌生态环境监测中心	AWA6228+	JC-1264	93.8	93.6	94.0	AWA6223-F	FZ-0735
360123	2023	360123200020	安义县文峰塔	115.5462	28.8401	沿河大道	1800	20	4	2	2	南昌市安义县文峰塔	0.5	4	17	17	9	0	7	67.1	63.4	51.4	47.2	94.1	44.1	6.9	江西省南昌生态环境监测中心	AWA6228+	JC-1076	93.8	93.7	94.0	AWA6223-F	FZ-0735
360123	2023	360123200021	板溪村委会	115.5388	28.8349	桥南路	1300	25	2	2	3	南昌市安义县板溪村委会	0.6	4	17	17	9	2	59	64.4	68.4	60.0	53.0	79.2	45.7	5.8	江西省南昌生态环境监测中心	AWA6228+	JC-1264	93.8	93.6	94.0	AWA6223-F	FZ-0735
360123	2023	360123200022	国家电网	115.6245	28.8749	东阳大道	700	40	4	2	2	南昌市安义县国家电网	0.2	4	17	10	48	35	121	71.5	75.2	64.0	57.8	92.2	53.6	6.6	江西省南昌生态环境监测中心	AWA6228+	JC-1265	93.8	93.7	94.0	AWA6223-F	FZ-0735
360124	2023	360124200001	寺前万家	116.2231	28.4048	腾飞大道	5767	45	6	2	2	南昌市进贤县寺前万家	0.9	3	14	10	29	42	175	66.3	71.0	62.2	51.8	77.7	45.3	6.8	江西省南昌生态环境监测中心	AWA6228+	JC-1264	93.8	93.4	94.0	AWA6223-F	FZ-0737
360124	2023	360124200002	康盛阳光尊邸	116.2345	28.4008	北一路	1354	24	4	2	6	南昌市进贤县康盛阳光尊邸	1.2	3	14	10	33	0	27	60.9	64.6	56.4	51.0	83.2	46.2	5.1	江西省南昌生态环境监测中心	AWA6228+	JC-1265	93.8	93.9	94.0	AWA6221A	FZ-0556
360124	2023	360124200003	碧桂园青岚府	116.2289	28.3971	滨湖大道	2313	32	4	2	3	南昌市进贤县碧桂园青岚府	1.1	3	14	11	3	0	24	64.4	65.6	59.4	54.2	85.2	48.7	4.9	江西省南昌生态环境监测中心	AWA6228+	JC-1264	93.8	93.6	94.0	AWA6223-F	FZ-0737
360124	2023	360124200004	德丰洗涤	116.2185	28.3972	青阳大道	1144	60	4	2	2	南昌市进贤县圆通科技大门北侧	0.7	3	14	9	57	9	58	65.7	67.2	60.6	56.6	90.0	53.0	4.4	江西省南昌生态环境监测中心	AWA6228+	JC-1264	93.8	93.7	94.0	AWA6223-F	FZ-0737
360124	2023	360124200005	民和镇政府	116.2427	28.3819	民和路	1925	32	4	2	3	南昌市进贤县民和镇政府大门北	2.3	3	14	15	53	4	110	63.4	65.6	58.2	52.0	86.3	44.7	5.5	江西省南昌生态环境监测中心	AWA6228+	JC-1264	93.8	93.6	94.0	AWA6223-F	FZ-0737
360124	2023	360124200006	进贤华影院	116.2566	28.3820	滨湖大道	4185	45	4	2	2	南昌市进贤县进贤华影院西侧	1.3	3	14	15	17	4	151	63.8	67.4	61.0	52.6	83.6	46.5	5.5	江西省南昌生态环境监测中心	AWA6228+	JC-1264	93.8	93.3	94.0	AWA6223-F	FZ-0737
360124	2023	360124200007	二中初中部	116.2475	28.3784	人民大道	3000	45	6	2	2	南昌市进贤县二中初中部大门北侧	3.1	3	14	15	22	18	112	69.9	71.8	61.8	52.8	97.5	47.1	7.2	江西省南昌生态环境监测中心	AWA6228+	JC-1265	93.8	93.6	94.0	AWA6221A	FZ-0556
360124	2023	360124200008	民和六小	116.2427	28.3745	董源路	3200	24	4	2	6	南昌市进贤县民和六小入口西	2.7	3	14	9	17	4	126	66.4	69.0	62.2	55.4	89.0	50.0	5.3	江西省南昌生态环境监测中心	AWA6228+	JC-1076	93.8	93.6	94.0	AWA6223-F	FZ 0735
360124	2023	360124200009	宏舟实业	116.2366	28.3727	进贤大道	4398	60	4	2	2	南昌市进贤县宏舟实业大门对面东	0.8	3	14	9	55	40	280	72.6	74.0	68.2	61.0	94.9	53.8	5.3	江西省南昌生态环境监测中心	AWA6228+	JC-1077	93.8	93.7	94.0	AWA6223-F	FZ-0737

表3　2023年南昌市道路交通噪声昼间例行监测

行政区划代码	监测年度	点位编码	测点名称	测点经度	测点纬度	路段名称	路段长度（m）	道路总宽度（m）	机动车车道数	车道类别	道路等级	测点参照物	路段覆盖人口（万人）	月	日	时	分	大型车车流量	中小型车车流量	Leq	L10	L50	L90	最大值	最小值	标准差(SD)	监测站名	监测仪器型号	监测仪器编号	监测前校准值	监测后校准值	声校准器测量声压值	声校准仪器型号	声校准仪器编号
360124	2023	360124200010	天集广场	116.2605	28.3627	进贤大道	5430	60	4	2	2	南昌市进贤县天集广场天虹对面	2.7	3	14	11	6	24	144	67.4	70.2	63.4	58.2	90.0	53.3	4.7	江西省南昌生态环境监测中心	AWA6228+	JC-1076	93.8	93.9	94.0	AWA6223-F	FZ-0737
360124	2023	360124200011	洪福肠衣	116.2839	28.3492	东外环路	1680	45	2	2	2	南昌市进贤县洪福肠衣大门东72m	0.2	3	14	14	1	24	46	70.6	73.0	58.8	51.8	93.2	49.0	8.2	江西省南昌生态环境监测中心	AWA6228+	JC-1077	93.8	94.0	94.0	AWA6223-F	FZ-0737
360124	2023	360124200012	向阳服饰	116.2103	28.3828	潘李路（西一路）民和路	1680	18	2	2	6	南昌市进贤县向阳服饰大门对面南	0.7	3	14	9	6	8	26	65.0	66.4	51.4	48.8	93.5	47.8	6.5	江西省南昌生态环境监测中心	AWA6228+	JC-1264	93.8	93.5	94.0	AWA6223-F	FZ-0737
360124	2023	360124200013	彩帛针织	116.2157	28.3946	青岚大道	3520	60	4	2	2	南昌市进贤县彩帛针织大门南45m	0.9	3	14	9	59	8	45	66.7	69.4	55.6	48.2	85.6	44.4	8.1	江西省南昌生态环境监测中心	AWA6228+	JC-1265	93.8	93.6	94.0	AWA6221A	FZ-0556
360124	2023	360124200014	茂昌饲料	116.2213	28.3841	燕曹路	1945	18	2	2	6	南昌市进贤县茂昌饲料大门北侧20m	0.8	3	14	9	12	9	43	66.6	69.6	56.8	50.4	90.3	47.1	7.3	江西省南昌生态环境监测中心	AWA6228+	JC-1265	93.8	93.7	94.0	AWA6221A	FZ-0556
360124	2023	360124200015	近湖佳苑南	116.2355	28.3950	贤士三路	1550	24	4	2	6	南昌市进贤县近湖佳苑南	1.3	3	14	11	5	12	48	65.6	68.2	60.6	53.0	84.5	46.2	5.7	江西省南昌生态环境监测中心	AWA6228+	JC-1265	93.8	93.6	94.0	AWA6221A	FZ-0556
360124	2023	360124200016	特种纸业	116.2398	28.3882	中山大道	2880	60	8	2	2	南昌市进贤县特种纸业入口	1.7	3	14	15	55	8	108	64.9	68.6	61.8	55.0	87.2	47.0	5.2	江西省南昌生态环境监测中心	AWA6228+	JC-1265	93.8	93.7	94.0	AWA6221A	FZ-0556
360124	2023	360124200017	吴家村委会	116.2399	28.3685	中山大道	4390	60	6	2	2	南昌市进贤县吴家村入口北30m	2.1	3	14	10	0	5	145	60.2	64.4	54.2	48.6	85.3	44.6	5.7	江西省南昌生态环境监测中心	AWA6228+	JC-1076	93.8	94.0	94.0	AWA6223-F	FZ-0735
360124	2025	360124200018	康盛阳光城	116.2466	28.3665	云桥路	2653	45	4	2	2	南昌市进贤县康盛阳光城东南侧	2.4	3	14	10	26	18	114	66.1	69.8	62.0	54.0	83.8	46.8	6.0	江西省南昌生态环境监测中心	AWA6228+	JC-1077	93.8	94.0	94.0	AWA6223-F	FZ-0737
360124	2023	360124200019	北际塑胶	116.2508	28.3486	云桥路	2340	45	2	2	2	南昌市进贤县北际塑胶大门南侧	1.6	3	14	11	16	13	201	66.3	68.6	63.8	59.2	87.0	53.9	3.8	江西省南昌生态环境监测中心	AWA6228+	JC-1076	93.8	94.2	94.0	AWA6223-F	FZ-0735
360124	2023	360124200020	好日子橱柜	116.2520	28.3750	嘉禾路	1365	18	4	2	6	南昌市进贤县好日子橱柜前	2.6	3	14	9	11	9	63	67.8	68.8	62.4	57.6	93.2	52.7	4.8	江西省南昌生态环境监测中心	AWA6228+	JC-1077	93.8	93.6	94.0	AWA6223-F	FZ-0737
360124	2023	360124200021	进贤县交通局	116.2546	28.3631	胜利路	3325	32	2	2	3	南昌市进贤县交通局大门南侧	2.9	3	14	10	32	11	109	66.1	69.4	61.6	55.2	85.2	49.7	5.5	江西省南昌生态环境监测中心	AWA6228+	JC-1076	93.8	94.1	94.0	AWA6223-F	FZ-0735
360124	2023	360124200022	县人民医院	116.2707	28.3712	港边站路	1425	60	2	2	2	南昌市进贤县人民医院大门北侧	1.2	3	14	15	22	41	156	64.2	67.8	60.2	53.7	94.7	50.3	6.6	江西省南昌生态环境监测中心	AWA6228+	JC-1076	93.8	94.0	94.0	AWA6223-F	FZ-0735
360124	2023	360124200023	进出口检验站	116.2625	28.3385	医科大道	1425	60	4	2	2	南昌市进贤县进出口检验站北20米	0.4	3	14	14	46	12	87	59.4	63.2	56.8	50.8	84.3	46.9	6.7	江西省南昌生态环境监测中心	AWA6228+	JC-1076	93.8	94.2	94.0	AWA6223-F	FZ-0735
360124	2023	360124200024	中交路桥	116.2725	28.3420	创业大道	1425	60	4	2	2	南昌市进贤县中交路桥大门前	0.8	3	14	14	9	9	28	65.8	66.0	52.4	41.6	87.4	33.6	9.4	江西省南昌生态环境监测中心	AWA6228+	JC-1076	93.8	94.0	94.0	AWA6223-F	FZ-0735
/																																		

表4　2023年南昌市道路交通噪声夜间例行监测

行政区划代码	监测年度	点位编码	测点名称	测点经度	测点纬度	路段名称	路段长度（m）	道路总宽度（m）	机动车车道数	车道类别	道路等级	测点参照物	路段覆盖人口（万人）	月	日	时	分	大型车车流量	中小型车车流量	Leq	L10	L50	L90	最大值	最小值	标准差(SD)	监测站名	监测仪器型号	监测仪器编号	监测前校准值	监测后校准值	声校准器测量声压值	声校准仪器型号	声校准仪器编号
360100	2023	360111200118	艾溪湖北路Ⅰ	115.9950	28.7130	艾溪湖北路	6965	30	4	2	2	南昌市高新区中兴和园	0.3572	8	31	22	16	16	680	64.9	68.4	62.4	53.4	78.7	46.0	5.9	江西省南昌生态环境监测中心	AWA6228+	JC-1315	93.8	94.2	94.0	AWA6223-F	FZ-0735
360100	2023	360103200062	八一大道Ⅱ号	115.9005	28.6699	八一大道	3031	50	10	2	2	南昌市西湖区赣江宾馆	0.2425	9	1	4	1	1	193	63.2	66.6	59.8	52.8	88.6	43.8	5.4	江西省南昌生态环境监测中心	AWA6228+	JC-1318	93.8	93.7	94.0	AWA6223-F	FZ-0737
360100	2023	360103200096	北京路Ⅰ号	115.9096	28.6771	北京西路	2171	41	6	2	2	南昌市西湖区工商银行北京西路支行	0.1737	9	1	3	54	0	116	62.1	66.0	58.0	48.4	78.8	42.2	6.6	江西省南昌生态环境监测中心	AWA6228+	JC-1265	93.8	93.9	94.0	AWA6223-F	FZ-0737
360100	2023	360111200098	北京路Ⅲ号	115.9497	28.6779	北京东路	4821	48	6	2	2	南昌市青山湖区江西省肿瘤医院	0.3857	8	31	23	43	18	640	67.1	70.4	65.8	55.8	81.3	47.0	5.7	江西省南昌生态环境监测中心	AWA6228+	JC-1315	93.8	94.0	94.0	AWA6223-F	FZ-0735
360100	2023	360111200112	昌东大道Ⅰ号	115.9939	28.7005	昌东大道	5631	77	6	2	2	南昌市高新区武警水电二路总队医院	0.2779	8	31	22	49	22	161	57.2	61.9	53.5	49.1	71.9	43.8	4.7	江西省南昌生态环境监测中心	AWA6228	JC-JS030	93.8	93.4	94.0	AWA6223-F	FZ-0735
360100	2023	360111200114	昌东大道Ⅲ号	115.9755	28.6484	昌东大道	4087	36	4	2	2	南昌市青山湖区城东花园	0.223	9	18	22	0	59	375	65.8	68.8	62.0	54.6	95.7	48.7	5.4	江西省南昌生态环境监测中心	AWA6228+	JC-1076	93.8	93.5	94.0	AWA6223-F	FZ-0735
360100	2023	360111200115	昌东大道Ⅳ号	115.9746	28.6185	昌东大道	5264	36	6	2	2	南昌市青山湖区雪津南昌啤酒公司	0.4211	9	18	22	51	30	171	62.0	65.2	57.6	51.6	79.0	46.2	5.3	江西省南昌生态环境监测中心	AWA6228+	JC-1076	93.8	93.7	94.0	AWA6223-F	FZ-0735
360100	2023	360103200109	昌南大道Ⅰ号	115.8651	28.6158	昌南大道	6465	40	6	2	2	南昌市西湖区象湖水产场	0.117	9	12	1	26	72	102	69.5	73.0	64.0	56.6	94.3	47.1	6.3	江西省南昌生态环境监测中心	AWA6228+	JC-1265	93.8	93.8	94.0	AWA6223-F	FZ-0737
360100	2023	360111200111	昌南大道Ⅲ号	115.9423	28.6007	昌南大道	6569	52	6	2	2	南昌市青山湖区京川村	0.2922	9	18	23	48	64	48	65.1	68.2	60.4	55.8	82.7	53.0	4.7	江西省南昌生态环境监测中心	AWA6228+	JC-1315	93.8	93.9	94.0	AWA6223-F	FZ-0735
360100	2023	360111200015	丰和大道Ⅱ号	115.8697	28.7086	丰和北大道	3844	45	6	2	2	南昌市红谷滩区南昌日报社	0.2334	9	12	3	10	11	51	61.8	64.0	51.2	42.8	91.3	38.4	13.7	江西省南昌生态环境监测中心	AWA6228+	JC-1077	93.8	94.0	94.0	AWA6223-F	FZ-0736
360100	2023	360111200016	丰和大道Ⅲ号	115.8533	28.6964	丰和中大道	4217	56	6	2	2	南昌市红谷滩区丰和小区	0.3374	9	12	2	26	8	49	60.0	64.6	51.0	44.0	76.0	41.6	7.6	江西省南昌生态环境监测中心	AWA6228+	JC-1077	93.8	94.0	94.0	AWA6223-F	FZ-0736
360100	2023	360112200017	丰和大道Ⅳ号	115.8261	28.6461	丰和南大道	5327	56	6	2	2	南昌市红谷滩区中环医院	0.2841	9	12	1	20	14	63	69.6	73.4	61.4	53.4	89.6	49.0	7.2	江西省南昌生态环境监测中心	AWA6228+	JC-1319	93.8	93.7	94.0	AWA6223-F	FZ-0736
360100	2023	360103200052	抚河路Ⅰ号	115.8794	28.6760	抚河北路	1536	24	6	2	3	南昌市西湖区独一处粥城	0.1229	9	12	4	42	0	42	62.0	66.0	50.0	43.0	78.9	40.2	8.7	江西省南昌生态环境监测中心	AWA6228+	JC-1265	93.8	93.8	94.0	AWA6223-F	FZ-0737
360100	2023	360103200053	抚河路Ⅱ号	115.8879	28.6674	抚河中路	1707	24	6	2	3	南昌市西湖区永新汽车装璜维修中心	0.1366	9	12	3	18	0	51	64.1	68.0	60.8	47.4	79.7	40.3	7.5	江西省南昌生态环境监测中心	AWA6228+	JC-1318	93.8	93.5	94.0	AWA6223-F	FZ-0737
360100	2023	360104200054	抚河路Ⅲ号	115.8961	28.6523	抚河南路	2369	30	6	2	2	南昌市青云谱区省建材大市场	0.1895	9	11	23	3	3	251	65.8	69.6	63.6	53.4	75.5	42.6	6.1	江西省南昌生态环境监测中心	AWA6228+	JC-1318	93.8	93.7	94.0	AWA6223-F	FZ-0737
360100	2023	360103200041	抚生路Ⅰ号	115.8742	28.6627	抚生路	1028	31	4	2	3	南昌市西湖区卓品骄阳住宅小区	0.0822	9	11	22	17	1	85	59.1	63.0	54.6	48.6	82.3	44.8	5.3	江西省南昌生态环境监测中心	AWA6228+	JC-1265	93.8	93.7	94.0	AWA6223-F	FZ-0737
360100	2023	360103200042	抚生路Ⅱ号	115.8664	28.6491	抚生南路	2508	31	4	2	3	南昌市西湖区大塘村农民公寓	0.2006	9	11	23	45	8	131	62.4	65.6	58.0	51.4	79.8	48.3	5.4	江西省南昌生态环境监测中心	AWA6228+	JC-1318	93.8	93.8	94.0	AWA6223-F	FZ-0737
360100	2023	360103200043	抚生路Ⅲ号	115.8534	28.6291	抚生南路	2710	31	4	2	3	南昌市西湖区抚生路小学	0.1084	9	12	2	22	3	31	58.8	59.8	46.6	42.6	79.8	40.4	7.1	江西省南昌生态环境监测中心	AWA6228+	JC-1318	93.8	93.8	94.0	AWA6223-F	FZ-0737
360100	2023	360111200020	赣江大道Ⅰ号	115.8801	28.7119	赣江北大道	3409	18	4	2	2	南昌市红谷滩区绿宝竹木业公司	0.0884	9	12	3	43	0	10	62.5	63.4	51.2	47.2	95.6	40.3	5.9	江西省南昌生态环境监测中心	AWA6228+	JC-1077	93.8	94.2	94.0	AWA6223-F	FZ-0736
360100	2023	360102200021	赣江大道Ⅱ号	115.8625	28.6915	赣江中大道	4007	36	6	2	2	南昌市红谷滩区南昌广播电视中心	0.1603	9	12	1	51	0	17	60.1	63.8	53.4	42.0	86.1	38.8	8.2	江西省南昌生态环境监测中心	AWA6228+	JC-1077	93.8	94.1	94.0	AWA6223-F	FZ-0736
360100	2023	360112200023	赣江大道Ⅳ号	115.8473	28.6618	赣江南大道	5379	40	4	2	2	南昌市红谷滩区赣江新天地网球场	0.161	9	12	1	9	6	53	65.6	70.2	57.2	48.8	85.7	46.9	7.8	江西省南昌生态环境监测中心	AWA6228+	JC-1077	93.8	93.9	94.0	AWA6223-F	FZ-0736
360100	2023	360111200082	高新大道Ⅱ号	115.9561	28.7025	高新大道	6104	50	6	2	2	南昌市青山湖区江西天一药业公司	0.3454	9	1	0	23	12	112	59.4	63.2	55.8	48.1	76.7	44.3	5.8	江西省南昌生态环境监测中心	AWA6228	JC-JS030	93.8	93.5	94.0	AWA6223-F	FZ-0735
360100	2023	360111200086	高新大道Ⅵ号	115.9518	28.6353	高新南大道	8837	50	6	2	2	南昌市青山湖区石桥村	0.4579	9	18	23	6	8	218	57.2	60.8	55.0	48.0	75.4	42.7	4.7	江西省南昌生态环境监测中心	AWA6228+	JC-1315	93.8	93.4	94.0	AWA6223-F	FZ-0735
360100	2023	360104200108	广州路Ⅱ号	115.9435	28.6233	广州路	5946	39	4	2	2	南昌市青山湖区万溪村	0.3363	9	18	23	42	8	82	58.1	61.8	54.0	47.3	72.2	42.4	6.0	江西省南昌生态环境监测中心	AWA6228	JC-JS030	93.8	93.9	94.0	AWA6223-F	FZ-0735
360100	2023	360103200048	洪城路Ⅱ号	115.8871	28.6586	洪城路	3909	34	8	2	2	南昌市西湖区东方巴黎大厦	0.3127	9	11	23	2	49	343	69.7	73.6	66.6	57.8	88.4	49.8	6.0	江西省南昌生态环境监测中心	AWA6228+	JC-1265	93.8	93.7	94.0	AWA6223-F	FZ-0737
360100	2023	360102200067	洪都大道Ⅰ号	115.9177	28.6987	洪都北大道	4463	44	6	2	2	南昌市东湖区江西省地震局	0.357	9	1	2	23	47	59	69.5	73.8	63.8	52.0	85.0	44.4	8.0	江西省南昌生态环境监测中心	AWA6228+	JC-1265	93.8	93.7	94.0	AWA6223-F	FZ-0737
360100	2023	360103200070	洪都大道Ⅳ号	115.9225	28.6632	洪都中大道	2245	44	6	2	2	南昌市青山湖区隆鑫广场小区	0.1796	9	11	23	36	30	198	65.1	69.1	61.7	58.4	79.6	55.5	4.7	江西省南昌生态环境监测中心	AWA6228	JC-JS030	93.8	93.9	94.0	AWA6223-F	FZ-0735
360100	2023	360111200095	火炬大街Ⅰ号	115.9601	28.6942	火炬大街	2815	35	4	2	2	南昌市青山湖区百大集团物流中心	0.2252	9	1	0	15	52	547	58.4	62.0	55.2	47.2	72.0	41.8	5.6	江西省南昌生态环境监测中心	AWA6228+	JC-1076	93.8	93.7	94.0	AWA6223-F	FZ-0735
360100	2023	360111200104	解放路Ⅱ号	115.9357	28.6554	解放西路	3993	35	6	2	2	南昌市青山湖区南昌公交修理二厂	0.3194	9	12	0	25	19	195	64.2	66.4	62.2	58.2	85.0	54.2	3.3	江西省南昌生态环境监测中心	AWA6228+	JC-1315	93.8	93.9	94.0	AWA6223-F	FZ-0735
360100	2023	360111200106	解放路Ⅳ号	115.9638	28.6449	解放东路	4976	42	4	2	2	南昌市青山湖区沈桥村	0.3981	9	18	22	25	8	156	61.4	65.0	58.7	52.9	74.6	48.6	4.9	江西省南昌生态环境监测中心	AWA6228	JC-JS030	93.8	93.8	94.0	AWA6223-F	FZ-0735
360100	2023	360111200025	锦江路Ⅰ号	115.8724	28.7179	锦江路	1432	16	4	2	3	南昌市红谷滩区凤凰花园	0.1146	9	12	3	51	0	10	53.9	52.0	49.0	48.0	83.7	42.3	3.0	江西省南昌生态环境监测中心	AWA6228+	JC-1319	93.8	94.0	94.0	AWA6223-F	FZ-0736
360100	2023	360111200089	京东大道Ⅱ号	115.9683	28.7069	京东大道	6442	40	4	2	2	南昌市青山湖区江西丰采丽印刷包装公司	0.5154	9	1	0	56	18	219	56.9	60.8	48.4	45.6	73.8	44.3	5.9	江西省南昌生态环境监测中心	AWA6228+	JC-1076	93.8	93.7	94.0	AWA6223-F	FZ-0735
360100	2023	360111200091	京东大道Ⅳ号	115.9627	28.6685	京东南大道	3559	40	4	2	2	南昌市青山湖区世纪风情住宅小区	0.1924	8	31	23	34	1	108	52.9	56.3	50.2	46.0	67.4	42.5	4.6	江西省南昌生态环境监测中心	AWA6228	JC-JS030	93.8	93.6	94.0	AWA6223-F	FZ-0735
360100	2023	360104200064	井岗山大道Ⅱ	115.9069	28.6489	井冈山大道	4692	50	8	2	2	南昌市青云谱区深圳农产品批发市场	0.3754	9	12	0	39	3	209	61.5	65.8	56.2	47.0	85.3	41.9	6.8	江西省南昌生态环境监测中心	AWA6228+	JC-1076	93.8	93.9	94.0	AWA6223-F	FZ-0735
360100	2023	360103200050	九洲大街Ⅰ号	115.8580	28.6415	九洲大街	1366	44	6	2	3	南昌市西湖区观洲村	0.1093	9	12	0	35	1	46	60.4	63.6	56.6	53.2	82.1	50.5	4.1	江西省南昌生态环境监测中心	AWA6228+	JC-1318	93.8	93.6	94.0	AWA6223-F	FZ-0737

表4　2023年南昌市道路交通噪声夜间例行监测

行政区划代码	监测年度	点位编码	测点名称	测点经度	测点纬度	路段名称	路段长度（m）	道路总宽度（m）	机动车车道数	车道类别	道路等级	测点参照物	路段覆盖人口（万人）	月	日	时	分	大型车车流量	中小型车车流量	Leq	L10	L50	L90	最大值	最小值	标准差(SD)	监测站名	监测仪器型号	监测仪器编号	监测前校准值	监测后校准值	声校准器测量声压值	声校准仪器型号	声校准仪器编号
360100	2023	360103200051	九洲大街Ⅱ号	115.8706	28.6319	九洲大街	2077	44	6	2	3	南昌市西湖区南昌动物园	0.1662	9	12	0	29	11	51	65.4	67.4	51.0	45.4	84.6	43.5	8.6	江西省南昌生态环境监测中心	AWA6228+	JC-1265	93.8	93.7	94.0	AWA6223-F	FZ-0737
360100	2023	360111200010	庐山南大道Ⅰ	115.8461	28.7366	庐山南大道	1964	46	4	2	2	南昌市青山湖区昌北电信分公司	0.1571	9	20	2	41	22	130	55.5	59.6	52.2	45.2	68.1	42.1	6.2	江西省南昌生态环境监测中心	AWA6228+	JC-1077	93.8	93.7	91.0	AWA6223-F	FZ-0736
360100	2023	360111200012	庐山南大道Ⅲ	115.8594	28.7063	庐山南大道	3865	35	4	2	2	南昌市红谷滩区工商银行昌北支行	0.3092	9	12	3	10	8	76	57.1	60.4	53.8	52.6	68.7	52.1	3.2	江西省南昌生态环境监测中心	AWA6228+	JC-1319	93.8	94.2	94.0	AWA6223-F	FZ-0736
360100	2023	360104200066	南莲路Ⅰ号	115.9183	28.6140	南莲路	2439	32	4	2	2	南昌市青云谱区熊坊小学	0.1951	9	19	0	20	30	164	70.2	73.7	64.5	53.4	92.1	46.9	7.5	江西省南昌生态环境监测中心	AWA6228	JC-JS030	93.8	93.5	94.0	AWA6223 F	FZ-0735
360100	2023	360112200029	南斯友好路Ⅰ	115.8348	28.6749	南斯友好路	3132	24	4	2	2	南昌市红谷滩区省二手车市场	0.2506	9	12	2	2	28	127	57.1	56.8	44.0	40.4	81.4	38.9	6.8	江西省南昌生态环境监测中心	AWA6228+	JC-1319	93.8	94.1	91.0	AWA6223-F	FZ-0736
360100	2023	360112200034	前湖大道Ⅰ号	115.8214	28.6612	前湖大道	4034	82	6	2	2	南昌市红谷滩区南昌航空大学红角洲校区	0.2656	9	12	0	32	0	72	63.3	67.8	54.6	46.6	82.4	40.2	7.9	江西省南昌生态环境监测中心	AWA6228+	JC-1077	93.8	93.5	94.0	AWA6223-F	FZ-0736
360100	2023	360111200007	青岚大道Ⅰ号	115.8199	28.7505	青岚大道	1651	16	4	2	3	南昌市青山湖区昌北经济适用房	0.066	9	20	2	8	6	12	60.9	60.2	56.6	47.2	74.8	41.6	5.9	江西省南昌生态环境监测中心	AWA6228+	JC-1077	93.8	93.6	94.0	AWA6223-F	FZ-0736
360100	2023	360111200008	青岚大道Ⅱ号	115.8198	28.7351	青岚大道	3466	16	4	2	3	南昌市青山湖区江西三星气龙公司	0.2053	9	20	1	41	17	32	59.5	61.0	51.0	47.4	83.7	46.1	5.7	江西省南昌生态环境监测中心	AWA6228+	JC-1319	93.8	94.0	91.0	AWA6223-F	FZ-0736
360100	2023	360102200059	青山路Ⅰ号	115.9243	28.7138	青山北路	2288	22	4	2	3	南昌市东湖区江西强力工量刃具公司	0.183	9	1	1	39	3	35	60.1	64.2	52.2	44.6	75.3	41.4	7.1	江西省南昌生态环境监测中心	AWA6228+	JC-1265	93.8	93.8	94.0	AWA6223-F	FZ-0737
360100	2023	360102200060	青山路Ⅱ号	115.9086	28.7028	青山南路	2632	35	6	2	2	南昌市东湖区百货大楼青山路店	0.2106	9	1	1	45	0	166	63.1	66.8	60.0	52.0	80.4	45.5	5.6	江西省南昌生态环境监测中心	AWA6228+	JC-1318	93.8	93.8	94.0	AWA6223-F	FZ-0737
360100	2023	360103200074	孺子路Ⅰ号	115.8849	28.6748	孺子路	776	23	4	2	3	南昌市西湖区南昌华云中医院	0.0621	9	12	4	46	0	74	63.2	67.6	56.4	49.2	78.1	45.2	6.8	江西省南昌生态环境监测中心	AWA6228+	JC-1318	93.8	93.9	94.0	AWA6223-F	FZ-0737
360100	2023	360103200075	孺子路Ⅱ号	115.8932	28.6751	孺子路	1020	25	4	2	3	南昌市西湖区金世家橱柜	0.0816	9	19	0	56	9	177	70.8	74.2	66.2	57.0	91.7	47.6	6.5	江西省南昌生态环境监测中心	AWA6228+	JC-1265	93.8	93.8	94.0	AWA6223-F	FZ-0737
360100	2023	360104200077	三店西路Ⅱ号	115.9061	28.6338	三店西路	1503	20	4	2	3	南昌市青云谱区青云明珠小区	0.1202	9	12	1	34	1	16	56.2	58.6	49.4	46.4	77.6	42.8	5.0	江西省南昌生态环境监测中心	AWA6228+	JC-1076	93.8	93.6	94.0	AWA6223-F	FZ-0735
360100	2023	360111200078	上海路Ⅰ号	115.9356	28.6880	上海北路	2771	20	4	2	3	南昌市青山湖区南昌大学附属中学	0.2217	9	1	2	55	2	35	53.1	57.4	43.0	39.2	71.6	36.9	6.9	江西省南昌生态环境监测中心	AWA6228+	JC-1076	93.8	93.7	94.0	AWA6223-F	FZ-0735
360100	2023	360111200079	上海路Ⅱ号	115.9361	28.6705	上海路	2438	29	4	2	3	南昌市青山湖区南昌新时代妇科医院	0.195	9	1	3	27	0	225	62.3	66.2	59.0	52.0	78.8	46.7	5.3	江西省南昌生态环境监测中心	AWA6228+	JC-1315	93.8	93.6	94.0	AWA6223-F	FZ-0735
360100	2023	360102200028	世贸路Ⅱ号	115.8525	28.6853	世贸路	2036	18	4	2	3	南昌市红谷滩区南昌市人民政府	0.1629	9	12	2	39	0	16	59.4	63.8	54.6	45.6	74.0	41.2	6.5	江西省南昌生态环境监测中心	AWA6228+	JC-1319	93.8	94.1	94.0	AWA6223-F	FZ-0736
360100	2023	360111200002	双港大街Ⅱ号	115.8312	28.7456	双港西大街	3884	32	6	2	2	南昌市青山湖区神基科技南昌公司	0.2227	9	20	2	14	0	9	54.1	56.8	51.6	49.6	73.0	40.7	3.6	江西省南昌生态环境监测中心	AWA6228+	JC-1319	93.8	93.7	94.0	AWA6223-F	FZ-0736
360100	2023	360111200003	双港大街Ⅲ号	115.8647	28.7464	双港东大街	4175	50	6	2	2	南昌市青山湖区华东交大南区专家楼	0.1918	9	20	3	10	6	20	56.0	59.4	54.0	45.8	66.0	40.8	5.5	江西省南昌生态环境监测中心	AWA6228+	JC-1077	93.8	93.7	94.0	AWA6223-F	FZ-0736
360100	2023	360111200101	顺外路Ⅱ号	115.9481	28.6649	顺外路	5342	17	4	2	2	南昌市青山湖区肖坊村民委员会	0.4274	9	11	23	17	10	155	61.1	63.0	54.6	48.4	88.4	42.1	5.8	江西省南昌生态环境监测中心	AWA6228+	JC-1076	93.8	93.5	94.0	AWA6223-F	FZ-0735
360100	2023	360103200044	桃花路Ⅰ号	115.8790	28.6622	桃花路	1138	19	4	2	3	南昌市西湖区远东国际花园	0.091	9	11	22	20	4	206	66.1	69.4	63.2	55.8	81.3	50.6	5.2	江西省南昌生态环境监测中心	AWA6228+	JC-1318	93.8	93.6	94.0	AWA6223-F	FZ-0737
360100	2023	360103200045	桃花路Ⅱ号	115.8764	28.6422	桃花南路	3209	38	6	2	3	南昌市西湖区坝上新村	0.1938	9	11	23	40	3	71	62.4	66.0	55.6	48.2	80.4	43.6	6.7	江西省南昌生态环境监测中心	AWA6228+	JC-1265	93.8	93.7	94.0	AWA6223-F	FZ-0737
360100	2023	360103200046	桃花路Ⅲ号	115.8667	28.6233	桃花南路	1940	38	6	2	3	南昌市西湖区十里庙村	0.1552	9	12	1	35	7	101	61.3	64.6	53.0	43.4	78.9	38.8	7.9	江西省南昌生态环境监测中心	AWA6228+	JC-1318	93.8	93.9	94.0	AWA6223-F	FZ-0737
360100	2023	360121200116	天祥大道Ⅰ号	116.0219	28.6982	天祥大道	5830	21	4	2	2	南昌市高新区南昌工业职业技术学院	0.3413	8	31	22	31	7	180	65.6	66.2	57.6	51.4	91.6	46.6	5.9	江西省南昌生态环境监测中心	AWA6228+	JC-1317	93.8	93.8	94.0	AWA6223-F	FZ-0737
360100	2023	360112200036	祥云大道Ⅰ号	115.8030	28.6356	祥云大道	8314	48	6	2	2	南昌市红谷滩区中铁16局4公司南昌项目部	0.4047	9	11	23	53	30	80	63.1	67.2	52.2	44.8	84.2	42.5	8.4	江西省南昌生态环境监测中心	AWA6228+	JC-1077	93.8	93.6	94.0	AWA6223-F	FZ-0736
360100	2023	360102200057	象山路Ⅰ号	115.8861	28.6825	象山北路	1367	18	4	2	3	南昌市东湖区南昌市第一医院	0.1094	9	19	1	38	1	56	60.5	64.4	55.4	49.4	81.7	46.3	5.6	江西省南昌生态环境监测中心	AWA6228+	JC-1318	93.8	93.8	94.0	AWA6223-F	FZ-0737
360100	2023	360103200058	象山路Ⅱ号	115.8897	28.6712	象山南路	1715	12	2	2	3	南昌市西湖区象山广场	0.1372	9	19	0	58	0	66	57.0	60.0	51.2	44.4	78.9	40.9	6.0	江西省南昌生态环境监测中心	AWA6228+	JC-1318	93.8	93.8	94.0	AWA6223-F	FZ-0737
360100	2023	360112200031	学府大道Ⅰ号	115.7945	28.6536	学府大道	7414	44	6	2	2	南昌市红谷滩区南昌大学红角洲校区	0.4461	9	12	0	4	3	42	58.8	61.6	47.0	43.8	77.4	41.6	7.1	江西省南昌生态环境监测中心	AWA6228+	JC-1319	93.8	93.9	94.0	AWA6223-F	FZ-0736
360100	2023	360102200037	沿江大道Ⅰ号	115.8892	28.7011	沿江北大道	6135	16	4	2	2	南昌市东湖区滨江宾馆	0.2454	9	12	4	2	4	126	69.7	73.2	60.6	49.2	88.6	39.4	8.9	江西省南昌生态环境监测中心	AWA6228+	JC-1318	93.8	93.7	94.0	AWA6223-F	FZ-0737
360100	2023	360103200038	沿江大道Ⅱ号	115.8685	28.6710	沿江快速路	2926	18	6	2	1	南昌市西湖区海关大楼	0.117	9	27	1	7	7	221	66.3	70.0	64.4	56.0	81.5	48.4	5.4	江西省南昌生态环境监测中心	AWA6228+	JC-1317	93.8	93.8	94.0	AWA6223-F	FZ-0737
360100	2023	360103200040	沿江大道Ⅳ号	115.8421	28.6340	沿江快速路	4410	20	6	2	1	南昌市西湖区净业寺	0.1764	9	12	2	25	29	160	63.4	66.6	61.6	54.2	83.5	44.7	4.8	江西省南昌生态环境监测中心	AWA6228+	JC-1265	93.8	93.7	94.0	AWA6223-F	FZ-0737
360100	2023	360102200073	阳明路Ⅱ号	115.9084	28.6915	阳明东路	2597	39	6	2	2	南昌市东湖区贤士湖住宅小区	0.2078	9	1	2	31	0	132	61.6	66.0	57.8	48.6	79.1	37.4	6.7	江西省南昌生态环境监测中心	AWA6228+	JC-1318	93.8	93.9	94.0	AWA6223-F	FZ-0737
360100	2023	360104200056	迎宾大道Ⅱ号	115.9028	28.6253	迎宾大道	5919	30	4	2	2	南昌市青云谱区江铃汽车股份公司	0.4735	9	12	1	42	8	106	62.0	66.5	55.6	43.5	80.2	38.1	8.7	江西省南昌生态环境监测中心	AWA6228	JC-JS030	93.8	94.1	94.0	AWA6223-F	FZ-0735
360100	2023	360111200005	玉屏大街Ⅱ号	115.8178	28.7264	玉屏西大街	3587	16	4	2	2	南昌市青山湖区南昌凯马柴油机公司	0.195	9	20	1	9	126	107	69.4	74.4	60.0	51.6	85.7	48.5	8.3	江西省南昌生态环境监测中心	AWA6228+	JC-1319	93.8	93.6	94.0	AWA6223-F	FZ-0736
360100	2023	360121200121	紫阳大道Ⅱ号	116.0140	28.6784	紫阳大道	6031	68	6	2	2	南昌市高新区蓝天学院瑶湖校区	0.3458	8	31	23	59	8	248	61.7	64.6	58.0	50.4	79.0	44.5	5.5	江西省南昌生态环境监测中心	AWA6228+	JC-1265	93.8	93.8	94.0	AWA6223-F	FZ-0737
360100	2023	360121200123	艾溪湖南路Ⅰ	115.9932	28.6706	艾溪湖南路	4610	30	6	2	3	南昌市高新区中骏蓝湾香郡	0.2369	8	31	23	1	7	378	59.7	63.2	55.8	47.6	73.2	42.2	5.9	江西省南昌生态环境监测中心	AWA6228+	JC-1076	93.8	93.6	94.0	AWA6223-F	FZ-0735

表4　2023年南昌市道路交通噪声夜间例行监测

行政区划代码	监测年度	点位编码	测点名称	测点经度	测点纬度	路段名称	路段长度（m）	道路总宽度（m）	机动车车道数	车道类别	道路等级	测点参照物	路段覆盖人口（万人）	月	日	时	分	大型车车流量	中小型车车流量	Leq	L10	L50	L90	最大值	最小值	标准差(SD)	监测站名	监测仪器型号	监测仪器编号	监测前校准值	监测后校准值	声校准器测量声压值	声校准仪器型号	声校准仪器编号
360100	2023	360121200124	艾溪湖南路Ⅱ	116.0180	28.6707	艾溪湖南路	2896	30	6	2	3	南昌市高新区小邹石村	0.1421	9	1	0	44	3	49	61.8	65.6	49.6	42.2	81.1	39.5	8.5	江西省南昌生态环境监测中心	AWA6228+	JC-1318	93.8	93.6	94.0	AWA6223-F	FZ-0737
360100	2023	360102200125	爱国路Ⅰ号	115.8884	28.6971	爱国路	3383	10	2	2	3	南昌市东湖区爱国路省军区	0.2706	9	12	3	57	1	3	62.9	56.0	44.0	42.0	95.0	40.7	6.9	江西省南昌生态环境监测中心	AWA6228+	JC-1265	93.8	93.9	94.0	AWA6223-F	FZ-0737
360100	2023	360112200126	白玉兰路Ⅰ号	115.7863	28.7100	白玉兰路	2915	20	4	2	3	南昌市新建区万科金域传奇	0.153	9	19	23	24	3	43	56.3	57.6	51.6	50.0	77.2	47.8	3.6	江西省南昌生态环境监测中心	AWA6228+	JC-1319	93.8	93.6	94.0	AWA6223-F	FZ-0736
360100	2023	360104200127	博学路Ⅰ号	115.9362	28.6298	博学路	1662	20	4	2	3	南昌市青云谱区博泰生命树小区	0.099	9	18	23	34	0	31	58.8	60.8	49.8	45.2	78.9	42.2	6.4	江西省南昌生态环境监测中心	AWA6228+	JC-1076	93.8	93.5	94.0	AWA6223-F	FZ-0735
360100	2023	360111200128	昌安路Ⅰ号	115.9713	28.6299	昌安路	3841	15	4	2	3	南昌市青山湖区华奥印务有限公司	0.2268	9	18	23	1	1	8	56.6	57.0	54.7	53.7	72.1	52.1	3.0	江西省南昌生态环境监测中心	AWA6228	JC-JS030	93.8	93.5	94.0	AWA6223-F	FZ-0735
360100	2023	360112200129	城运大道Ⅰ号	115.8104	28.6032	城运大道	8480	40	6	2	3	南昌市红谷滩区南昌光年装饰有限公司	0.1808	9	18	23	46	0	68	58.1	61.6	48.4	43.6	76.2	41.6	6.9	江西省南昌生态环境监测中心	AWA6228+	JC-1265	93.8	93.7	94.0	AWA6223-F	FZ-0737
360100	2023	360121200130	创新一路Ⅰ号	115.9979	28.6989	创新一路	4743	10	2	2	3	南昌市高新区千僖同业	0.2808	8	31	22	46	0	157	57.1	60.2	50.8	46.2	72.8	41.6	5.5	江西省南昌生态环境监测中心	AWA6228+	JC-1315	93.8	93.9	94.0	AWA6223-F	FZ-0735
360100	2023	360111200131	东泰大道Ⅰ号	115.9696	28.6382	东泰大道	6157	18	4	2	3	南昌市青山湖区江苏皮尔萨管业	0.4161	9	18	22	9	8	58	56.3	60.0	52.8	49.4	69.9	47.1	4.1	江西省南昌生态环境监测中心	AWA6228+	JC-1315	93.8	93.5	94.0	AWA6223-F	FZ-0735
360100	2023	360102200132	二七北路Ⅰ号	115.9135	28.6805	二七北路	3021	16	4	2	3	南昌市东湖区东来顺饭庄对面	0.2417	9	1	3	17	0	33	58.4	60.6	46.6	38.4	77.4	35.9	8.6	江西省南昌生态环境监测中心	AWA6228+	JC-1318	93.8	93.8	94.0	AWA6223-F	FZ-0737
360100	2023	360102200133	福州路Ⅰ号	115.9085	28.6866	福州路	1539	15	4	2	3	南昌市东湖区金昌利大厦对面	0.1231	9	1	3	11	0	91	52.4	54.0	49.4	48.0	71.5	46.2	3.0	江西省南昌生态环境监测中心	AWA6228+	JC-1265	93.8	93.9	94.0	AWA6223-F	FZ-0737
360100	2023	360111200134	富大有路Ⅰ号	115.9503	28.7323	富大有路	6324	30	6	2	3	南昌市青山湖区万科金域滨江	0.133	8	31	22	3	23	331	64.9	68.1	60.6	57.7	83.5	51.7	4.6	江西省南昌生态环境监测中心	AWA6228	JC-JS030	93.8	93.9	94.0	AWA6223-F	FZ-0735
360100	2023	360121200135	富大有路Ⅱ号	115.9963	28.7248	富大有路	6741	40	6	2	3	南昌市高新区白云驾校	0.0708	8	31	22	8	42	1237	69.9	72.8	67.4	61.4	84.4	56.0	4.5	江西省南昌生态环境监测中心	AWA6228+	JC-1076	93.8	93.6	94.0	AWA6223-F	FZ-0735
360100	2023	360111200136	高新七路Ⅰ号	115.9714	28.7039	高新七路	2180	15	4	2	3	南昌市青山湖区南昌外国语高新学校	0.1744	9	1	0	54	0	22	60.8	64.4	50.0	45.6	78.5	42.6	7.3	江西省南昌生态环境监测中心	AWA6228+	JC-1315	93.8	93.7	94.0	AWA6223-F	FZ-0735
360100	2023	360112200137	工业大道Ⅰ号	115.7854	28.6991	工业大道	2977	40	6	2	2	南昌市新建区南昌顺发机动车检测有限公司	0.1902	9	19	23	13	38	74	59.6	58.8	49.0	44.0	79.9	39.3	6.4	江西省南昌生态环境监测中心	AWA6228+	JC-1077	93.8	93.7	94.0	AWA6223-F	FZ-0736
360100	2023	360111200138	海棠南路Ⅰ号	115.8086	28.7311	海棠南路	2451	28	4	2	3	南昌市青山湖区江西财大麦庐校区	0.1481	9	20	1	35	2	25	59.4	61.6	48.4	43.4	88.9	41.1	7.2	江西省南昌生态环境监测中心	AWA6228+	JC-1077	93.8	93.7	94.0	AWA6223-F	FZ-0736
360100	2023	360112200139	黄家湖东路Ⅰ	115.8239	28.7106	黄家湖东路	2355	40	6	2	2	南昌市新建区奥克斯盛世华庭南门	0.1395	9	20	0	33	6	92	63.4	64.4	52.2	45.6	85.2	43.0	7.7	江西省南昌生态环境监测中心	AWA6228+	JC-1319	93.8	94.1	94.0	AWA6223-F	FZ-0736
360100	2023	360111200140	黄家湖西路Ⅰ	115.8045	28.7162	黄家湖西路	4188	40	6	2	2	南昌市青山湖区汉港凯旋城	0.2062	9	20	1	1	0	32	60.6	64.4	54.8	47.2	86.4	38.4	6.7	江西省南昌生态环境监测中心	AWA6228+	JC-1077	93.8	93.6	94.0	AWA6223-F	FZ-0736
360100	2023	360111200141	火炬二路Ⅰ号	115.9592	28.6973	火炬二路	2765	10	2	2	3	南昌市青山湖区大唐烧烤	0.2212	9	1	0	18	0	22	58.1	62.0	49.2	42.4	79.1	39.6	7.3	江西省南昌生态环境监测中心	AWA6228+	JC-1315	93.8	93.9	94.0	AWA6223-F	FZ-0735
360100	2023	360111200142	火炬三路Ⅰ号	115.9665	28.7048	火炬三路	2109	10	4	2	3	南昌市青山湖区汉庭酒店	0.1687	9	11	22	28	0	58	59.1	62.8	54.6	49.4	74.7	47.5	5.1	江西省南昌生态环境监测中心	AWA6228+	JC-1076	93.8	93.6	94.0	AWA6223-F	FZ-0735
360100	2023	360111200143	火炬五路Ⅰ号	115.9726	28.7184	火炬五路	2914	18	4	2	3	南昌市青山湖区百利精密刀具（南昌）有限公司	0.2331	9	1	1	22	0	17	55.3	55.4	51.2	49.8	74.0	48.2	3.4	江西省南昌生态环境监测中心	AWA6228+	JC-1076	93.8	93.7	94.0	AWA6223-F	FZ-0735
360100	2023	360111200144	金港路Ⅰ号	115.8820	28.7688	金港路	4441	24	4	2	3	南昌市青山湖区江西华盛钢铁实业有限公司	0.2278	9	20	4	30	14	45	53.6	57.8	50.6	46.4	70.8	43.1	7.6	江西省南昌生态环境监测中心	AWA6228+	JC-1077	93.8	93.6	94.0	AWA6223-F	FZ-0736
360100	2023	360111200145	金山大道Ⅰ号	115.9168	28.7736	金山大道	8983	50	6	2	2	南昌市青山湖区洪城警苑小区	0.0961	9	12	4	29	22	1	67.3	68.2	53.6	45.0	97.5	39.0	9.0	江西省南昌生态环境监测中心	AWA6228+	JC-1077	93.8	94.2	94.0	AWA6223-F	FZ-0736
360100	2023	360111200146	京安路Ⅰ号	115.9611	28.6715	京安路	1525	21	4	2	3	南昌市青山湖区水之韵养生馆	0.122	8	31	23	36	36	412	54.2	56.6	47.8	44.8	78.7	42.0	4.8	江西省南昌生态环境监测中心	AWA6228+	JC-1076	93.8	93.6	94.0	AWA6223-F	FZ-0735
360100	2023	360111200147	经开大道Ⅰ号	115.8766	28.7721	经开大道	7598	34	6	2	2	南昌市青山湖区南昌志航无缝钢管球墨铸铁管公司	0.302	9	12	5	23	17	36	62.3	59.6	47.0	40.8	96.3	38.8	7.6	江西省南昌生态环境监测中心	AWA6228+	JC-1319	93.8	94.0	94.0	AWA6223-F	FZ-0736
360100	2023	360112200148	景德镇街Ⅰ号	115.7911	28.6153	景德镇街	3551	27	6	2	3	南昌市红谷滩区腾龙学校	0.142	9	18	23	53	0	20	56.2	56.4	50.4	46.8	75.7	43.5	4.4	江西省南昌生态环境监测中心	AWA6228+	JC-1318	93.8	93.9	94.0	AWA6223-F	FZ-0737
360100	2023	360112200149	九龙大道Ⅰ号	115.7912	28.6053	九龙大道	7583	75	12	2	2	南昌市红谷滩区绿地国际博览中心	0.2443	9	18	23	3	37	57	68.3	70.8	57.2	49.6	86.1	41.7	8.1	江西省南昌生态环境监测中心	AWA6228+	JC-1265	93.8	93.7	94.0	AWA6223-F	FZ-0737
360100	2023	360111200150	梅林大道Ⅰ号	115.8741	28.7606	梅林大道	2919	28	4	2	3	南昌市青山湖区海立电器	0.2335	9	20	3	19	5	17	59.7	58.4	55.4	54.8	85.8	53.9	2.5	江西省南昌生态环境监测中心	AWA6228+	JC-1319	93.8	93.9	94.0	AWA6223-F	FZ-0736
360100	2023	360112200151	梅岭大道Ⅰ号	115.7456	28.6829	梅岭大道	3956	34	6	2	2	南昌市新建区湾里江西中医大学科技学院	0.1727	9	11	23	11	2	37	58.3	61.0	52.6	43.8	84.2	39.4	6.6	江西省南昌生态环境监测中心	AWA6228+	JC-1319	93.8	93.9	94.0	AWA6223-F	FZ-0736
360100	2023	360111200152	民营大道Ⅰ号	115.9500	28.7161	民营大道	2084	30	4	2	3	南昌市青山湖区星辉村安置房	0.1667	9	1	2	25	1	12	54.9	55.0	44.4	40.4	79.9	39.1	6.0	江西省南昌生态环境监测中心	AWA6228+	JC-1076	93.8	93.6	94.0	AWA6223-F	FZ-0735
360100	2023	360111200153	民营大道Ⅱ号	115.9498	28.7019	民营大道	1991	30	4	2	3	南昌市青山湖区车漆世界钣喷中心	0.1593	9	1	1	54	3	29	56.4	55.4	40.8	36.2	80.6	34.2	7.8	江西省南昌生态环境监测中心	AWA6228+	JC-1076	93.8	93.7	94.0	AWA6223-F	FZ-0735
360100	2023	360112200154	明矾路Ⅰ号	115.8023	28.7089	明矾路	2054	26	6	2	3	南昌市新建区城西市容环境服务站	0.1643	9	19	23	49	0	30	57.6	61.4	50.0	41.0	72.3	37.0	7.4	江西省南昌生态环境监测中心	AWA6228+	JC-1077	93.8	93.6	94.0	AWA6223-F	FZ-0736
360100	2023	360112200155	南龙蟠街Ⅰ号	115.7901	28.5827	南龙蟠街	2481	28	6	2	3	南昌市红谷滩区九龙湖南地铁站东	0.0992	9	18	23	5	0	75	56.6	59.8	51.2	45.8	81.7	42.7	5.4	江西省南昌生态环境监测中心	AWA6228+	JC-1318	93.8	93.5	94.0	AWA6223-F	FZ-0737
360100	2023	360111200156	青山湖大道Ⅰ	115.9453	28.7072	青山湖大道	5117	29	6	2	2	南昌市青山湖区二七王食府	0.4094	9	1	1	59	26	22	61.6	62.4	49.8	41.2	83.5	38.7	8.2	江西省南昌生态环境监测中心	AWA6228+	JC-1315	93.8	93.6	94.0	AWA6223-F	FZ-0735
360100	2023	360111200157	青山湖大道Ⅱ	115.9445	28.6683	青山湖大道	3930	29	6	2	2	南昌市青山湖区南铁世纪花园	0.3144	9	11	23	10	10	60	60.3	64.0	55.0	48.4	75.6	42.7	6.0	江西省南昌生态环境监测中心	AWA6228+	JC-1315	93.8	93.6	94.0	AWA6223-F	FZ-0735
360100	2023	360104200158	墅溪路Ⅰ号	115.9286	28.6474	墅溪路	2715	26	6	2	3	南昌市青云谱区保利和光小区入口	0.2172	9	12	0	52	2	41	60.7	64.1	51.3	44.3	77.0	41.2	7.7	江西省南昌生态环境监测中心	AWA6228	JC-JS030	93.8	94.0	94.0	AWA6223-F	FZ-0735

表4　2023年南昌市道路交通噪声夜间例行监测

行政区划代码	监测年度	点位编码	测点名称	测点经度	测点纬度	路段名称	路段长度（m）	道路总宽度（m）	机动车车道数	车道类别	道路等级	测点参照物	路段覆盖人口（万人）	月	日	时	分	大型车车流量	中小型车车流量	Leq	L10	L50	L90	最大值	最小值	标准差(SD)	监测站名	监测仪器型号	监测仪器编号	监测前校准值	监测后校准值	声校准器测量声压值	声校准仪器型号	声校准仪器编号
360100	2023	360112200159	双马石路Ⅰ号	115.7266	28.7018	双马石路	1861	16	4	2	3	南昌市新建区湾里天安财险	0.0981	9	11	22	26	6	58	63.8	65.4	57.8	51.2	91.2	47.5	5.4	江西省南昌生态环境监测中心	AWA6228+	JC-1319	93.8	93.6	94.0	AWA6223-F	FZ-0736
360100	2023	360112200160	天宁路Ⅰ号	115.7294	28.6945	天宁路	3808	26	4	2	3	南昌市新建区湾里金佳·云舒花城	0.184	9	11	22	53	0	45	65.1	68.4	60.2	55.4	89.0	46.8	5.2	江西省南昌生态环境监测中心	AWA6228+	JC-1077	93.8	93.9	94.0	AWA6223-F	FZ-0736
360100	2023	360112200161	文化大道Ⅰ号	115.8196	28.7002	文化大道	3577	34	4	2	2	南昌市新建区米罗小区南门	0.2106	9	20	0	27	5	103	64.4	66.0	56.4	46.8	70.6	42.8	7.5	江西省南昌生态环境监测中心	AWA6228+	JC-1077	93.8	93.7	94.0	AWA6223-F	FZ-0736
360100	2023	360112200162	武功山大道Ⅰ	115.7358	28.6359	武功山大道	9712	55	6	2	2	南昌市新建区联富花园A期7栋旁	0.561	9	18	22	12	84	153	63.4	67.8	55.0	44.6	84.6	40.1	8.7	江西省南昌生态环境监测中心	AWA6228+	JC-1317	93.8	93.8	94.0	AWA6223-F	FZ-0737
360100	2023	360104200163	新溪桥路Ⅰ号	115.9175	28.6397	新溪桥路	2238	24	6	2	3	南昌市青云谱区旺中旺生活超市洪都店	0.179	9	12	1	25	0	0	47.6	48.8	45.8	42.8	68.4	40.0	2.8	江西省南昌生态环境监测中心	AWA6228+	JC-1315	93.8	93.4	94.0	AWA6223-F	FZ-0735
360100	2023	360112200164	兴国路Ⅰ号	115.8037	28.6940	兴国路	4086	16	4	2	3	南昌市新建区警犬基地动物医院	0.2789	9	19	22	34	4	65	62.2	60.0	56.6	50.2	80.4	45.9	5.9	江西省南昌生态环境监测中心	AWA6228+	JC-1077	93.8	93.7	94.0	AWA6223-F	FZ-0736
360100	2023	360111200165	秀先路Ⅰ号	115.8977	28.7844	秀先路	2004	40	4	2	3	南昌市青山湖区南昌攀藤科技有限公司	0.048	9	12	4	43	1	5	48.6	49.4	43.8	41.8	67.2	38.8	3.6	江西省南昌生态环境监测中心	AWA6228+	JC-1319	93.8	94.1	94.0	AWA6223-F	FZ-0736
360100	2023	360121200166	瑶湖西大道Ⅰ	116.0335	28.6830	瑶湖西大道	3570	25	6	2	3	南昌市高新区昌东镇龚杏高新现代产业综合城	0.0902	8	31	23	21	19	65	62.7	66.6	54.2	48.2	80.2	42.2	7.0	江西省南昌生态环境监测中心	AWA6228+	JC-1317	93.8	93.8	94.0	AWA6223-F	FZ-0737
360100	2023	360121200167	瑶湖西大道Ⅱ	116.0437	28.7138	瑶湖西大道	4077	25	6	2	3	南昌市高新区昌东镇君江半岛	0.0956	8	31	22	17	19	80	63.5	65.2	60.0	57.4	91.4	54.0	3.2	江西省南昌生态环境监测中心	AWA6228+	JC-1265	93.8	93.7	94.0	AWA6223-F	FZ-0737
360100	2023	360121200168	瑶湖西一路Ⅰ	116.0232	28.6880	瑶湖西一路	2329	13	4	2	3	南昌市高新区昌东镇南昌工程学院	0.1863	8	31	23	14	2	24	61.3	62.4	47.0	43.0	82.3	39.7	8.0	江西省南昌生态环境监测中心	AWA6228+	JC-1265	93.8	93.8	94.0	AWA6223-F	FZ-0737
360100	2023	360111200169	英雄大道Ⅰ号	115.8694	28.7818	英雄大道	6005	24	6	2	3	南昌市青山湖区五联花园小区	0.2726	9	12	5	11	13	37	65.0	69.8	53.8	40.6	90.1	37.5	9.7	江西省南昌生态环境监测中心	AWA6228+	JC-1077	93.8	93.7	94.0	AWA6223-F	FZ-0736
360100	2023	360112200170	永强路Ⅰ号	115.7290	28.6404	永强路	4096	18	4	2	3	南昌市新建区太阳岛文具厂对面	0.3277	9	18	22	8	4	7	57.1	56.8	47.6	46.0	81.3	44.9	5.1	江西省南昌生态环境监测中心	AWA6228+	JC-1265	93.8	93.9	94.0	AWA6223-F	FZ-0737
360100	2023	360112200171	长堎大道Ⅰ号	115.7965	28.6894	长堎大道	3296	26	6	2	3	南昌市新建区江西新一建筑工程公司门口	0.195	9	19	22	43	3	52	57.9	61.0	56.4	51.8	74.9	47.4	3.6	江西省南昌生态环境监测中心	AWA6228+	JC-1319	93.8	94.0	94.0	AWA6223-F	FZ-0736
360100	2023	360112200172	长麦路Ⅰ号	115.8135	28.7041	长麦路	3100	24	6	2	3	南昌市新建区南昌新建一中	0.1991	9	19	23	59	3	34	57.1	60.6	51.4	44.8	82.9	41.4	6.0	江西省南昌生态环境监测中心	AWA6228+	JC-1319	93.8	93.9	94.0	AWA6223-F	FZ-0736
360100	2023	360112200173	长麦南路Ⅰ号	115.8145	28.6850	长麦南路	1581	24	6	2	3	南昌市新建区幸福里小区12栋旁	0.1265	9	19	22	8	4	78	64.4	66.0	61.8	58.4	89.2	48.2	3.6	江西省南昌生态环境监测中心	AWA6228+	JC-1077	93.8	93.7	94.0	AWA6223-F	FZ-0736
360100	2023	360112200174	长征西路Ⅰ号	115.8169	28.6829	长征西路	4123	15	6	2	3	南昌市新建区长征西路馨居雅苑对面	0.2202	9	19	22	0	6	98	82.1	66.2	60.6	55.0	113.7	44.3	6.4	江西省南昌生态环境监测中心	AWA6228+	JC-1319	93.8	93.6	94.0	AWA6223-F	FZ-0736
360100	2023	360112200175	招贤大道Ⅰ号	115.7339	28.7092	招贤大道	4292	38	6	2	2	南昌市新建区湾里COCO公馆	0.3019	9	11	22	15	20	230	63.9	67.2	60.2	55.6	83.7	52.0	4.7	江西省南昌生态环境监测中心	AWA6228+	JC-1077	93.8	93.7	94.0	AWA6223-F	FZ-0736
360100	2023	360104200176	朱桥东路Ⅰ号	115.9336	28.6108	朱桥东路	3151	30	4	2	3	南昌市青云谱区深圳农产品中心批发市场冻品区十号门	0.2141	9	19	0	11	3	17	62.4	66.4	57.8	52.2	80.4	48.4	5.3	江西省南昌生态环境监测中心	AWA6228+	JC-1076	93.8	93.6	94.0	AWA6223-F	FZ-0735
360121	2023	360121200001	三幼汇仁	115.9045	28.5712	工业一路	2552	12	4	2	3	南昌市南昌县三幼汇仁园区	0.16	2	23	22	0	0	37	57.6	61.2	49.4	43.2	79.0	40.0	6.8	江西省南昌生态环境监测中心	AWA6228+	JC-1264	93.8	93.7	94.0	AWA6021A	FZ-0736
360121	2023	360121200002	金沙幼儿园	115.8642	28.5667	金沙三路	7676	22	4	2	2	南昌市南昌县卓尔金沙幼儿园	0.19	2	14	22	0	2	98	66.0	70.4	59.0	49.8	82.4	44.5	7.4	江西省南昌生态环境监测中心	AWA6228+	JC-1076	93.8	93.8	94.0	AWA6021A	FZ-0735
360121	2023	360121200003	泰豪动漫学院	115.8743	28.5761	金沙二路	3360	16	4	2	2	南昌市南昌县泰豪动漫学院	0.29	2	14	22	30	6	264	63.3	66.0	60.8	55.0	80.0	47.0	4.4	江西省南昌生态环境监测中心	AWA6228+	JC-1076	93.8	93.8	94.0	AWA6021A	FZ-0735
360121	2023	360121200004	无端道医堂	115.8685	28.5830	东莲路	6117	26	6	2	2	南昌市南昌县新力幸福时光无端道医堂	0.17	2	15	0	35	0	25	53.5	56.0	42.8	34.8	73.0	31.7	7.9	江西省南昌生态环境监测中心	AWA6228+	JC-1077	93.8	93.8	94.0	AWA6021A	FZ-0737
360121	2023	360121200005	智慧树幼儿园	115.9221	28.5786	康莲路	1270	30	4	2	3	南昌市南昌县智慧树幼儿园	0.08	2	23	22	56	0	34	55.8	56.6	46.6	42.4	91.2	40.7	4.9	江西省南昌生态环境监测中心	AWA6228+	JC-1264	93.8	93.7	94.0	AWA6021A	FZ-0736
360121	2023	360121200006	百威雪津	115.8829	28.5615	金沙大道	18067	29	6	2	2	南昌市南昌县百威雪津（南昌）啤酒有限公司	0.35	2	14	23	35	20	272	68.6	72.4	64.6	52.4	84.2	43.8	7.5	江西省南昌生态环境监测中心	AWA6228+	JC-1076	93.8	93.8	94.0	AWA6021A	FZ-0735
360121	2023	360121200007	煌盛中央公园	115.9092	28.5687	迎宾中大道	6648	80	6	2	2	南昌市南昌县煌盛中央公园东门欧润油品	0.54	2	23	22	26	46	325	60.7	64.6	58.0	52.6	76.4	46.2	4.5	江西省南昌生态环境监测中心	AWA6228+	JC-1264	93.8	93.9	94.0	AWA6021A	FZ-0736
360121	2023	360121200008	绵竹幸福里	115.8601	28.6051	八月湖路	4305	24	8	2	2	南昌市南昌县绵竹幸福里七彩色地产	0.24	2	14	22	59	8	39	60.1	64.4	55.4	49.6	72.4	47.3	5.6	江西省南昌生态环境监测中心	AWA6228+	JC-1077	93.8	93.7	94.0	AWA6021A	FZ-0737
360121	2023	360121200009	景城名郡	115.8792	28.5886	芳草路	1979	12	4	2	3	南昌市南昌县景城名郡我爱我家	0.21	2	15	0	6	0	42	59.6	63.4	54.6	49.0	80.2	44.6	5.4	江西省南昌生态环境监测中心	AWA6228+	JC-1077	93.8	93.7	94.0	AWA6021A	FZ-0737
360121	2023	360121200010	棕榈学府	115.9140	28.5538	莲安南路	600	28	4	2	3	南昌市南昌县棕榈学府东新芝教	0.16	2	24	0	29	4	32	61.3	62.2	52.2	40.2	99.6	38.2	9.3	江西省南昌生态环境监测中心	AWA6228+	JC-1264	93.8	94.0	94.0	AWA6021A	FZ-0736
360121	2023	360121200011	五羊本田	115.9303	28.5792	南莲路	5335	46	6	2	2	南昌市南昌县鹿鼎爱丁堡五羊本田	0.48	2	23	23	24	57	389	65.5	68.8	63.8	55.8	77.6	50.1	4.9	江西省南昌生态环境监测中心	AWA6228+	JC-1264	93.8	94.1	94.0	AWA6021A	FZ-0736
360121	2023	360121200012	盐业包装	115.8881	28.5526	富山一路	4115	10	2	2	3	南昌市南昌县江西盐业包装有限公司与路对面	0.08	2	15	0	6	1	2	53.8	54.4	46.0	39.6	75.3	38.3	7.0	江西省南昌生态环境监测中心	AWA6228+	JC-1076	93.8	93.8	94.0	AWA6021A	FZ-0735
360121	2023	360121200013	中国民生银行	115.9274	28.5525	澄湖西路	3400	40	4	2	2	南昌市南昌县中国民生银行澄湖西路支行	0.58	2	14	23	33	0	170	60.8	63.0	55.6	49.6	79.3	44.3	5.4	江西省南昌生态环境监测中心	AWA6228+	JC-1265	93.8	93.8	94.0	AWA6021A	FZ-0736
360121	2023	360121200014	味思诺蛋糕	115.9375	28.5279	安宝大道	1100	40	6	2	3	南昌市南昌县银河城味思诺蛋糕	0.21	2	14	22	0	14	97	67.8	71.6	62.0	50.0	83.8	32.6	8.7	江西省南昌生态环境监测中心	AWA6228+	JC-1265	93.8	93.7	94.0	AWA6021A	FZ-0736
360121	2023	360121200015	居住主题公园	115.8838	28.5959	海嘉路	1561	12	4	2	3	南昌市南昌县南昌居住主题公园19栋西面	0.26	2	14	23	33	0	27	51.7	52.4	42.6	40.8	75.8	39.5	5.0	江西省南昌生态环境监测中心	AWA6228+	JC-1077	93.8	93.8	94.0	AWA6021A	FZ-0737
360121	2023	360121200016	新力象湖湾	115.8362	28.5944	东岳大道	4842	16	8	2	2	南昌市南昌县新力象湖湾南门京东家电	0.20	2	14	22	0	0	82	59.1	63.2	54.4	46.8	76.5	43.0	6.1	江西省南昌生态环境监测中心	AWA6228+	JC-1077	93.8	93.8	94.0	AWA6021A	FZ-0737
360121	2023	360121200017	梦里水乡	115.9198	28.5598	澄湖北路	2700	68	8	2	1	南昌市南昌县梦里水乡北门中国太平洋保险	0.24	2	23	23	58	8	206	62.5	67.0	56.4	48.2	76.7	41.8	6.8	江西省南昌生态环境监测中心	AWA6228+	JC-1264	93.8	93.7	94.0	AWA6021A	FZ-0736

表4　2023年南昌市道路交通噪声夜间例行监测

行政区划代码	监测年度	点位编码	测点名称	测点经度	测点纬度	路段名称	路段长度（m）	道路总宽度（m）	机动车车道数	车道类别	道路等级	测点参照物	路段覆盖人口（万人）	月	日	时	分	大型车车流量	中小型车车流量	Leq	L10	L50	L90	最大值	最小值	标准差(SD)	监测站名	监测仪器型号	监测仪器编号	监测前校准值	监测后校准值	声校准器测量声压值	声校准仪器型号	声校准仪器编号
360121	2023	360121200018	东新乡政府	115.8424	28.6049	东祥路	5020	24	6	2	2	南昌市南昌县东新乡政府东面	0.16	2	14	22	29	1	110	60.4	64.8	56.6	48.8	79.6	40.8	6.0	江西省南昌生态环境监测中心	AWA6228⁺	JC-1077	93.8	93.9	94.0	AWA6021A	FZ-0737
360121	2023	360121200019	力高君御华府	115.9280	28.5407	莲富路	1921	14	4	2	2	南昌市南昌县力高君御华府圣达文化艺术培训学校	0.12	2	14	23	2	0	69	58.3	62.4	53.8	47.8	72.8	43.8	5.5	江西省南昌生态环境监测中心	AWA6228⁺	JC-1265	93.8	93.8	94.0	AWA6021A	FZ-0736
360121	2023	360121200020	三志物流	115.8665	28.5445	富山大道	9919	20	6	2	2	南昌市南昌县富山大道290号南昌三志物流有限公司	0.21	2	15	0	37	22	96	62.4	66.6	52.8	46.6	78.0	42.4	7.5	江西省南昌生态环境监测中心	AWA6228⁺	JC-1076	93.8	93.8	94.0	AWA6021A	FZ-0735
360121	2023	360121200021	迪启博幼儿园	115.9395	28.5429	五一路	1253	10	2	2	2	南昌市南昌县迪启博幼儿园	0.08	2	14	22	32	0	64	60.5	63.0	55.8	48.4	82.7	33.4	5.9	江西省南昌生态环境监测中心	AWA6228⁺	JC-1265	93.8	93.8	94.0	AWA6021A	FZ-0736
360121	2023	360121200022	新力银湖湾	115.8890	28.5732	汇仁大道	6043	28	6	2	2	南昌市南昌县新力银湖湾中国电信	0.25	2	14	23	3	1	308	65.1	69.0	61.8	52.8	81.4	46.1	6.2	江西省南昌生态环境监测中心	AWA6228⁺	JC-1076	93.8	93.8	94.0	AWA6021A	FZ-0735
360121	2023	360121200023	烨达环保	115.8896	28.5388	富山二路	4154	9	2	2	3	南昌市南昌县江苏烨达环保驻江西生产基地	0.05	2	15	0	7	1	7	52.3	52.8	43.0	39.6	75.3	37.7	5.2	江西省南昌生态环境监测中心	AWA6228⁺	JC-1265	93.8	93.8	94.0	AWA6021A	FZ-0736
360121	2023	360121200024	宝江钢材	115.8862	28.5251	富山五路（汽车大道）	4972	30	6	2	2	南昌市南昌县宝江钢材股份有限公司	0.15	2	15	0	34	16	197	59.6	63.6	51.6	44.8	78.8	31.3	7.1	江西省南昌生态环境监测中心	AWA6228⁺	JC-1265	93.8	93.6	94.0	AWA6223-F	FZ-0736
360123	2023	360123200001	育苗幼儿园	115.5417	28.8475	北门路	735	10	1	1	3	南昌市安义县育苗幼儿园	0.5	4	17	22	15	0	6	56.0	55.2	44.2	40.6	83.8	38.0	6.0	江西省南昌生态环境监测中心	AWA6228⁺	JC-1315	93.8	94.2	94.0	AWA6221A	FZ-0556
360123	2023	360123200002	农税征收局	115.5437	28.8452	解放路	1233	20	2	2	3	南昌市安义县农业税征收管理局	0.6	4	17	22	40	1	31	59.4	62.8	53.0	47.8	80.2	45.0	5.7	江西省南昌生态环境监测中心	AWA6228⁺	JC-1077	93.8	94.1	94.0	AWA6223-F	FZ-0737
360123	2023	360123200003	前进村卫生所	115.5490	28.8427	东门路	1300	25	2	2	2	南昌市安义县龙津镇前进村卫生所	0.8	4	18	2	7	0	4	54.1	55.4	47.8	35.6	73.6	33.6	6.3	江西省南昌生态环境监测中心	AWA6228⁺	JC-1315	93.8	94.1	94.0	AWA6221A	FZ-0556
360123	2023	360123200004	鑫辉鞋业	115.5490	28.8426	人民路	1000	22	2	2	3	南昌市安义县鑫辉鞋业	0.9	4	17	22	45	0	3	54.5	57.2	48.2	44.0	74.4	39.1	5.2	江西省南昌生态环境监测中心	AWA6228⁺	JC-1264	93.8	94.1	94.0	AWA6223-F	FZ-0736
360123	2023	360123200005	金凤花园	115.5607	28.8465	延雄路	1000	24	4	2	2	南昌市安义县金凤花园管理处	0.9	4	17	23	20	0	19	57.1	60.8	49.6	43.4	79.0	39.7	6.6	江西省南昌生态环境监测中心	AWA6228⁺	JC-1315	93.8	94.1	94.0	AWA6221A	FZ-0556
360123	2023	360123200006	蓝调咖啡	115.5678	28.8474	达瑞路	1000	20	4	2	3	南昌市安义县蓝调咖啡	0.5	4	17	23	46	1	35	55.6	58.8	48.8	44.0	76.6	40.0	5.8	江西省南昌生态环境监测中心	AWA6228⁺	JC-1077	93.8	94.0	94.0	AWA6223-F	FZ-0737
360123	2023	360123200007	香榭大道	115.5733	28.8496	学府大道	1050	24	4	2	2	南昌市安义县香榭大道二期	0.3	4	17	23	49	0	16	48.4	51.0	40.6	37.8	71.1	35.1	5.4	江西省南昌生态环境监测中心	AWA6228⁺	JC-1264	93.8	94.1	94.0	AWA6223-F	FZ-0736
360123	2023	360123200008	伟星实业	115.5819	28.8544	凤凰大道	2064	32	4	2	2	南昌市安义县江西伟星实业	0.1	4	18	0	26	1	20	56.6	56.6	45.0	42.2	77.3	40.8	6.4	江西省南昌生态环境监测中心	AWA6228⁺	JC-1077	93.8	93.6	94.0	AWA6223-F	FZ-0737
360123	2023	360123200009	德旺不锈钢	115.5867	28.8459	引凤路	1575	25	4	2	2	南昌市安义县江西安邦德旺不锈钢	0.2	4	18	0	31	1	3	54.0	50.0	43.4	42.0	78.5	40.8	4.4	江西省南昌生态环境监测中心	AWA6228⁺	JC-1264	93.8	94.2	94.0	AWA6223-F	FZ-0736
360123	2023	360123200010	兰珠实业	115.5951	28.8589	孙虑成东路	2298	32	2	2	2	南昌市安义县南昌市兰珠实业	0.1	4	18	1	11	0	4	54.8	54.0	50.0	48.8	76.7	48.0	3.4	江西省南昌生态环境监测中心	AWA6228⁺	JC-1315	93.8	93.9	94.0	AWA6221A	FZ-0556
360123	2023	360123200011	勇居康酒店	115.6061	28.8612	凤凰东路	2104	40	2	2	2	南昌市安义县勇居康商务酒店	0.1	4	18	1	38	1	3	54.5	53.4	51.6	50.6	74.8	49.2	2.4	江西省南昌生态环境监测中心	AWA6228⁺	JC-1264	93.8	94.1	94.0	AWA6223-F	FZ-0736
360123	2023	360123200012	新凤玉石	115.5886	28.8650	创业大道	2251	36	4	2	2	南昌市安义县江西新凤微晶玉石公司	0.1	4	18	1	3	1	7	61.0	61.6	60.0	59.0	77.5	57.7	1.6	江西省南昌生态环境监测中心	AWA6228⁺	JC-1077	93.8	94.1	94.0	AWA6223-F	FZ-0737
360123	2023	360123200013	荣凯铝业	115.5964	28.8620	锦绣大道	2768	40	4	2	3	南昌市安义县荣凯铝业	0.1	4	18	1	10	11	18	61.8	63.2	50.2	47.8	84.7	45.8	6.4	江西省南昌生态环境监测中心	AWA6228⁺	JC-1264	93.8	93.9	94.0	AWA6223-F	FZ-0736
360123	2023	360123200014	和祥铝业	115.6028	28.8585	起秀路	1700	16	4	2	2	南昌市安义县江西和祥铝业	0.1	4	18	1	36	1	0	53.7	51.8	50.4	49.2	77.7	47.7	2.1	江西省南昌生态环境监测中心	AWA6228⁺	JC-1077	93.8	93.9	94.0	AWA6223-F	FZ-0737
360123	2023	360123200015	安义县华荟苑	115.5511	28.8498	建设路	1409	25	2	2	2	南昌市安义县华荟苑	0.5	4	17	23	16	0	40	58.4	61.6	53.2	46.4	81.0	41.0	6.0	江西省南昌生态环境监测中心	AWA6228⁺	JC-1077	93.8	94.2	94.0	AWA6223-F	FZ-0737
360123	2023	360123200016	安义县阳湖村	115.5538	28.8466	向阳路	2743	24	4	2	2	南昌市安义县龙津镇阳湖村前房文化活动中心	0.9	4	17	23	18	0	28	49.7	52.0	43.0	35.8	73.7	30.7	6.2	江西省南昌生态环境监测中心	AWA6228⁺	JC-1264	93.8	94.2	94.0	AWA6223-F	FZ-0736
360123	2023	360123200017	安义县清和园	115.5765	28.8466	前进路	4125	32	4	2	2	南昌市安义县清和园	0.6	4	17	23	51	0	26	56.7	59.8	53.8	50.4	75.7	48.8	3.6	江西省南昌生态环境监测中心	AWA6228⁺	JC-1315	93.8	94.2	94.0	AWA6221A	FZ-0556
360123	2023	360123200018	安义中学	115.5549	28.8420	文峰路	1639	32	6	2	2	南昌市安义县安义中学	0.9	4	17	22	49	0	84	62.7	67.2	57.4	46.8	76.5	39.5	7.5	江西省南昌生态环境监测中心	AWA6228⁺	JC-1315	93.8	93.7	94.0	AWA6221A	FZ-0556
360123	2023	360123200019	安义县滤城园	115.5908	28.8454	迎宾大道	2000	55	2	2	3	南昌市安义县滤城园农家饭庄	0.2	4	18	0	30	9	48	64.1	65.6	53.4	46.2	85.8	43.0	7.5	江西省南昌生态环境监测中心	AWA6228⁺	JC-1315	93.8	94.1	94.0	AWA6221A	FZ-0556
360123	2023	360123200020	安义县文峰塔	115.5462	28.8401	沿河大道	1800	20	4	2	2	南昌市安义县文峰塔	0.5	4	17	22	9	0	7	57.5	56.8	47.0	43.0	81.3	41.1	5.8	江西省南昌生态环境监测中心	AWA6228⁺	JC-1264	93.8	93.9	94.0	AWA6223-F	FZ-0736
360123	2023	360123200021	板溪村委会	115.5398	28.8349	桥南路	1300	25	2	2	3	南昌市安义县板溪村委会	0.6	4	17	22	2	2	30	61.0	64.2	55.2	46.8	76.8	38.7	6.6	江西省南昌生态环境监测中心	AWA6228⁺	JC-1077	93.8	93.9	94.0	AWA6223-F	FZ-0737
360123	2023	360123200022	国家电网	115.6245	28.8749	东阳大道	700	40	4	2	2	南昌市安义县国家电网	0.2	4	18	1	40	22	14	63.4	64.0	56.2	53.4	84.5	50.7	4.8	江西省南昌生态环境监测中心	AWA6228⁺	JC-1315	93.8	94.2	94.0	AWA6221A	FZ-0556
360124	2023	360124200001	寺前万家	116.2231	28.4048	腾飞大道	5767	45	6	2	2	南昌市进贤县寺前万家	0.9	3	29	0	24	31	11	63.1	67.0	52.4	44.2	80.9	34.9	8.8	江西省南昌生态环境监测中心	AWA6228⁺	JC-1264	93.8	94.0	94.0	AWA6223-F	FZ-0736
360124	2023	360124200002	康盛阳光尊邸	116.2345	28.4006	北一路	1354	24	4	2	6	南昌市进贤县康盛阳光尊邸	1.2	3	29	0	39	1	6	51.6	50.8	38.4	33.4	76.8	31.9	7.1	江西省南昌生态环境监测中心	AWA6228⁺	JC-1265	93.8	94.1	94.0	AWA6221A	FZ-0556
360124	2023	360124200003	碧桂园青岚府	116.2289	28.3971	滨湖大道	2313	32	4	2	3	南昌市进贤县碧桂园青岚府	1.1	3	29	0	43	0	2	48.2	46.4	33.8	30.4	74.6	28.8	6.9	江西省南昌生态环境监测中心	AWA6228⁺	JC-1077	93.8	94.1	94.0	AWA6223-F	FZ-0737
360124	2023	360124200004	德丰洗涤	116.2185	28.3972	青阳大道	1144	60	4	2	2	南昌市进贤县圆通科技大门北侧	0.7	3	29	1	0	1	2	46.7	48.2	41.5	36.4	63.4	34.0	5.2	江西省南昌生态环境监测中心	AWA6228⁺	JC-1265	93.8	93.3	94.0	AWA6221A	FZ-0556
360124	2023	360124200005	民和镇政府	116.2427	28.3819	民和路	1925	32	4	2	3	南昌市进贤县民和镇政府大门北	2.3	3	29	2	21	1	9	51.9	51.0	35.4	32.8	72.5	31.2	7.8	江西省南昌生态环境监测中心	AWA6228⁺	JC-1264	93.8	93.4	94.0	AWA6223-F	FZ-0736
360124	2023	360124200006	进贤华影院	116.2566	28.3820	滨湖大道	4185	45	4	2	2	南昌市进贤县进贤华影院西侧	1.3	3	29	2	38	0	41	59.4	62.8	51.8	44.4	78.6	40.7	6.9	江西省南昌生态环境监测中心	AWA6228⁺	JC-1265	93.8	94.1	94.0	AWA6221A	FZ-0556

表4　2023年南昌市道路交通噪声夜间例行监测

行政区划代码	监测年度	点位编码	测点名称	测点经度	测点纬度	路段名称	路段长度（m）	道路总宽度（m）	机动车车道数	车道类别	道路等级	测点参照物	路段覆盖人口（万人）	月	日	时	分	大型车车流量	中小型车车流量	Leq	L10	L50	L90	最大值	最小值	标准差(SD)	监测站名	监测仪器型号	监测仪器编号	监测前校准值	监测后校准值	声校准器测量声压值	声校准仪器型号	声校准仪器编号
360124	2023	360124200007	二中初中部	116.2475	28.3784	人民大道	3000	45	6	2	2	南昌市进贤县二中初中部大门北侧	3.1	3	29	2	25	0	12	51.1	51.8	39.8	33.6	73.3	27.4	7.2	江西省南昌生态环境监测中心	AWA6228+	JC-1077	93.8	94.1	94.0	AWA6223-F	FZ-0737
360124	2023	360124200008	民和六小	116.2427	28.3745	董源路	3200	24	4	2	6	南昌市进贤县民和六小入口西	2.7	3	29	3	7	0	15	55.9	56.2	38.6	30.8	81.6	28.4	9.7	江西省南昌生态环境监测中心	AWA6228+	JC-1265	93.8	93.5	94.0	AWA6221A	FZ-0556
360124	2023	360124200009	宏舟实业	116.2366	28.3727	进贤大道	4398	60	4	2	2	南昌市进贤县宏舟实业大门对面东	0.8	3	29	3	10	0	8	61.0	62.0	45.0	33.8	83.8	32.0	10.8	江西省南昌生态环境监测中心	AWA6228+	JC-1077	93.8	94.1	94.0	AWA6223-F	FZ-0737
360124	2023	360124200010	天集广场	116.2605	28.3627	进贤大道	5430	60	4	2	2	南昌市进贤县天集广场天虹对面	2.7	3	29	4	12	3	31	62.7	63.0	49.2	41.8	87.0	35.9	8.5	江西省南昌生态环境监测中心	AWA6228+	JC-1265	93.8	93.7	94.0	AWA6221A	FZ-0556
360124	2023	360124200011	洪福肠衣	116.2839	28.3492	东外环路	1680	45	2	2	2	南昌市进贤县洪福肠衣大门东72m	0.2	3	29	5	13	0	0	54.1	55.3	44.7	39.1	67.8	36.2	6.3	江西省南昌生态环境监测中心	AWA6228+	JC-1265	93.8	94.1	94.0	AWA6221A	FZ-0556
360124	2023	360124200012	向阳服饰	116.2103	28.3828	潘李路（西一路）民和路	1680	18	2	2	6	南昌市进贤县向阳服饰大门对面南	0.7	3	29	1	23	0	1	48.6	50.0	46.8	45.2	69.4	43.8	2.3	江西省南昌生态环境监测中心	AWA6228+	JC-1077	93.8	94.1	94.0	AWA6223-F	FZ-0737
360124	2023	360124200013	彩帛针织	116.2157	28.3946	青岚大道	3520	60	4	2	2	南昌市进贤县彩帛针织大门南45m	0.9	3	29	1	10	1	1	56.4	50.0	33.8	29.2	83.0	26.9	8.6	江西省南昌生态环境监测中心	AWA6228+	JC-1264	93.8	93.7	94.0	AWA6223-F	FZ-0736
360124	2023	360124200014	茂昌饲料	116.2213	28.3841	燕曹路	1945	18	2	2	6	南昌市进贤县茂昌饲料大门北侧20m	0.8	3	29	1	48	0	0	52.1	43.2	38.8	36.2	74.6	34.7	5.5	江西省南昌生态环境监测中心	AWA6228+	JC-1264	93.8	93.9	94.0	AWA6223-F	FZ-0736
360124	2023	360124200015	近湖佳苑南	116.2355	28.3950	贤士三路	1550	24	4	2	6	南昌市进贤县近湖佳苑南	1.3	3	29	1	55	0	0	42.1	42.6	35.4	31.0	73.6	27.5	4.7	江西省南昌生态环境监测中心	AWA6228+	JC-1265	93.8	94.2	94.0	AWA6221A	FZ-0556
360124	2023	360124200016	特种纸业	116.2398	28.3882	中山大道	2880	60	8	2	2	南昌市进贤县特种纸业入口	1.7	3	29	1	57	0	19	53.3	56.0	41.6	36.4	70.9	34.8	7.7	江西省南昌生态环境监测中心	AWA6228+	JC-1077	93.8	94.1	94.0	AWA6223-F	FZ-0737
360124	2023	360124200017	吴家村委会	116.2399	28.3685	中山大道	4390	60	6	2	2	南昌市进贤县吴家村入口北30m	2.1	3	29	3	35	3	3	49.5	49.0	35.0	30.0	72.8	27.7	8.0	江西省南昌生态环境监测中心	AWA6228+	JC-1264	93.8	93.6	94.0	AWA6223-F	FZ-0736
360124	2023	360124200018	康盛阳光城	116.2466	28.3665	云桥路	2653	45	4	2	2	南昌市进贤县康盛阳光城东南侧	2.4	3	29	3	39	0	11	53.7	54.2	38.4	36.0	76.8	33.4	7.6	江西省南昌生态环境监测中心	AWA6228+	JC-1265	93.8	93.3	94.0	AWA6221A	FZ-0556
360124	2023	360124200019	北际塑胶	116.2508	28.3486	云桥路	2340	45	2	2	2	南昌市进贤县北际塑胶大门南侧	1.6	3	29	4	7	1	4	55.5	52.2	46.4	45.2	82.7	43.7	4.5	江西省南昌生态环境监测中心	AWA6228+	JC-1264	93.8	94.2	94.0	AWA6223-F	FZ-0736
360124	2023	360124200020	好日子橱柜	116.2520	28.3750	嘉禾路	1365	18	4	2	6	南昌市进贤县好日子橱柜前	2.6	3	29	3	3	0	13	53.2	56.2	45.0	37.4	78.3	30.7	7.1	江西省南昌生态环境监测中心	AWA6228+	JC-1264	93.8	93.5	94.0	AWA6223-F	FZ-0736
360124	2023	360124200021	进贤县交通局	116.2546	28.3631	胜利路	3325	32	2	2	3	南昌市进贤县交通局大门南侧	2.9	3	29	3	42	0	24	61.0	63.4	47.2	34.6	81.4	29.9	10.7	江西省南昌生态环境监测中心	AWA6228+	JC-1077	93.8	94.1	94.0	AWA6223-F	FZ-0737
360124	2023	360124200022	县人民医院	116.2707	28.3712	港边站路	1425	60	2	2	2	南昌市进贤县人民医院大门北侧	1.2	3	29	4	16	0	5	49.8	52.6	41.0	39.2	74.3	38.0	5.7	江西省南昌生态环境监测中心	AWA6228+	JC-1077	93.8	94.1	94.0	AWA6223-F	FZ-0737
360124	2023	360124200023	进出口检验站	116.2625	28.3385	医科大道	1425	60	4	2	2	南昌市进贤县进出口检验站北20米	0.4	3	29	4	52	0	13	51.6	53.1	46.5	42.6	70.4	39.5	5.1	江西省南昌生态环境监测中心	AWA6228+	JC-1077	93.8	94.0	94.0	AWA6223-F	FZ-0737
360124	2023	360124200024	中交路桥	116.2725	28.3420	创业大道	1425	60	4	2	2	南昌市进贤县中交路桥大门前	0.8	3	29	5	7	3	19	50.7	52.6	38.4	32.9	71.5	28.6	5.6	江西省南昌生态环境监测中心	AWA6228+	JC-1264	93.8	94.1	94.0	AWA6223-F	FZ-0736
/																																		

报告编制　　　　复核　　　　审核　　　　签发

日　期 2023.10.25　　　　日期 2023.10.25　　　　日期 2023.10.25　　　　日期 2023.11.6
